# Imatinib in the Treatment of Colorectal Cancer—Molecular Targets, Preclinical Evidence and Clinical Limitations

**DOI:** 10.3390/molecules31213933

**Published:** 2026-11-01

**Authors:** Katarzyna Wanke, Mateusz Kciuk, Beata Marciniak, Renata Kontek

**Affiliations:** 1 Doctoral School of Exact and Natural Sciences, University of Lodz, 90-237 Lodz, Poland; katarzyna.wanke@edu.uni.lodz.pl; 2 Department of Molecular Biotechnology and Genetics, Faculty of Biology and Environmental Protection, University of Lodz, 90-237 Lodz, Poland; beata.marciniak@biol.uni.lodz.pl (B.M.); renata.kontek@biol.uni.lodz.pl (R.K.)

**Keywords:** imatinib, colorectal cancer, tyrosine kinase inhibitors, tumor microenvironment, targeted therapy

## Abstract

Colorectal cancer (CRC) remains a major therapeutic challenge, despite substantial advances in molecular characterization and targeted treatment. The heterogeneity of CRC and the development of treatment resistance highlight the need for novel therapeutic strategies and rational drug repurposing approaches. Imatinib, a well-established tyrosine kinase inhibitor, exerts pharmacological activity beyond ABL1/ABL2, targeting KIT, PDGFR, DDR1, and potentially LCK, which may contribute to distinct mechanisms of CRC progression. This review examines the emerging evidence on the potential role of imatinib in CRC, including its effects on tumor-cell proliferation and survival, invasion and metastasis, extracellular-matrix remodeling, tumor–stroma interactions, treatment resistance, and immune regulation. Preclinical studies indicate promising antitumor activity in selected molecular and biological contexts, particularly in KIT-positive tumors, PDGFR-rich stroma, mesenchymal/CMS4 tumors, and models characterized by aberrant DDR1 signaling. However, clinical evidence remains limited and does not currently support imatinib as a standard treatment for unselected CRC. Biomarker-guided patient selection, rational combination strategies, and tumor microenvironment-directed approaches may enhance its therapeutic potential. Further well-designed clinical studies are required to determine whether these biological effects can translate into meaningful clinical benefit.

## 1. Introduction

Despite significant progress in colorectal cancer (CRC) treatment, chemotherapy and currently available targeted therapies remain limited by treatment resistance, disease progression, and treatment-related adverse effects. These challenges continue to drive the search for new therapeutic strategies, including the repurposing of established anticancer drugs for indications beyond their original clinical applications. The development of targeted therapies has substantially changed modern oncology by enabling selective interference with molecular pathways involved in tumor development and progression. Among these approaches, tyrosine kinase inhibitors (TKIs) have become an important class of anticancer agents due to their ability to modulate signaling pathways involved in cell proliferation, survival, differentiation, migration, and other processes relevant to cancer biology [1].

Imatinib is one of the most extensively studied and clinically established TKIs. Its introduction represented an important milestone in targeted cancer therapy, demonstrating that inhibition of a specific molecular abnormality can translate into substantial clinical benefit [2]. Although its use has traditionally been associated with malignancies driven by defined tyrosine kinase abnormalities, the broader spectrum of its pharmacological activity has generated interest in its potential application beyond its original indications. This raises the possibility of reconsidering an established targeted agent in cancer types in which additional therapeutic strategies are needed.

CRC represents a particularly relevant setting in which to explore such therapeutic possibilities. Despite advances in molecular characterization and the increasing implementation of precision medicine, colorectal tumors remain highly heterogeneous, and therapeutic responses can vary considerably between patients. This molecular and biological complexity highlights the need to identify additional therapeutic vulnerabilities and to explore treatment strategies that may complement currently available approaches. In this context, the established pharmacological profile of imatinib provides a rationale for investigating its potential relevance in CRC [3].

In this review, we examine the current evidence concerning the potential role of imatinib in colorectal cancer and discuss the rationale for considering this established targeted agent in a new therapeutic context.

## 2. Literature Search and Study Selection

This article was prepared as a review of the preclinical and clinical evidence concerning the potential role of imatinib in colorectal cancer (CRC). A focused literature search was conducted in PubMed/MEDLINE, supplemented by searches of ClinicalTrials.gov and examination of the reference lists of relevant publications. Search terms included combinations of “imatinib,” “colorectal cancer,” “colon cancer,” “rectal cancer,” “ABL1,” “KIT,” “PDGFR,” “DDR1,” “LCK,” “tumor microenvironment,” “drug resistance,” and “clinical trial.”

Eligible sources included original in vitro and in vivo studies investigating imatinib activity in CRC models; experimental studies examining the biological roles of imatinib-sensitive kinases in CRC; clinical and translational studies evaluating imatinib in patients with CRC; and registered clinical trials assessing imatinib-based therapeutic strategies. Relevant review articles were also considered for background information and identification of additional primary studies. Studies investigating inhibitors other than imatinib were included when they provided mechanistic evidence relevant to the role of a particular kinase in CRC; however, their findings were not considered direct evidence of imatinib efficacy.

Sources were excluded when they did not address CRC biology, imatinib pharmacology, the molecular targets discussed in this review, or the clinical investigation of imatinib in a manner relevant to the scope of the article. The identified evidence was synthesized qualitatively, taking into account the experimental model, the type of intervention, the specificity of the mechanistic evidence, and the extent of clinical validation. This review did not involve a formal systematic-review screening protocol, quantitative meta-analysis, or formal risk-of-bias assessment.

## 3. Imatinib and Its Molecular Targets

Imatinib is a first-generation, adenosine triphosphate (ATP)-competitive type II tyrosine kinase inhibitor whose molecular target spectrum extends beyond the principal target—oncogenic breakpoint cluster region (BCR)/Abelson tyrosine-protein kinase 1 (ABL1) kinase [4,5]. Imatinib preferentially binds the inactive aspartate–phenylalanine–glycine-out (DFG-out) conformation of the kinase domain, thereby suppressing ABL1-mediated phosphorylation and downstream signaling involved in cell proliferation, survival, and transformation [6,7,8]. Imatinib also inhibits the closely related non-receptor tyrosine kinase Abelson-related gene (ABL2/ARG), although ABL2 is generally considered a secondary target relative to ABL1/BCR-ABL [9,10].

In addition, imatinib inhibits KIT proto-oncogene receptor tyrosine kinase (c-KIT/CD117) and platelet-derived growth factor receptor alpha (PDGFRα) and beta (PDGFRβ) [5], which explains its therapeutic activity in tumors driven by activating alterations in these kinases, most notably gastrointestinal stromal tumors (GISTs) and selected PDGFRA/B-dependent neoplasms [11,12,13].

The imatinib target spectrum further includes discoidin domain receptor 1 (DDR1), a collagen-activated receptor tyrosine kinase implicated in cell adhesion, extracellular-matrix signaling, proliferation, migration, and tumor progression [14,15,16,17].

Lymphocyte-specific protein tyrosine kinase (LCK), sarcoma proto-oncogene (Src)-family kinase essential for proximal T-cell receptor (TCR) signaling, has also been identified as an imatinib-sensitive kinase [18,19] and may represent a relevant off-target, particularly in immune cells, where its inhibition can modulate T-cell activation and regulatory T-cell survival [18,20].

Therefore, imatinib should be regarded as a multi-target kinase inhibitor rather than a strictly ABL1-selective agent, with its biological effects potentially reflecting the combined inhibition of ABL1/ABL2, KIT, PDGFRA/B, DDR1, and LCK.

The key pharmacophore elements of imatinib and their conceptual relationship to its type II, DFG-out binding mode and polypharmacology are illustrated in Figure 1.

## 4. Molecular Targets of Imatinib and Colorectal Cancer

### 4.1. ABL1

ABL1 (c-Abl) represents one of the molecular targets of imatinib that may be relevant to CRC, although its biological role in this cancer appears to be highly context-dependent. ABL1 is a ubiquitously expressed non-receptor tyrosine kinase involved in the regulation of cell adhesion, proliferation, differentiation, apoptosis, and responses to genotoxic stress. In one study, ABL1 expression was markedly increased in CRC tissues and cell lines, and its depletion in SW480 and HCT-116 cells inhibited proliferation, induced cell-cycle arrest, and enhanced apoptosis, while ABL1 downregulation in vivo reduced tumor progression. Mechanistically, ABL1 depletion was associated with increased cyclin-dependent kinase inhibitor 1B (P27) and decreased cyclin D1 expression, together with increased B-cell lymphoma 2 (BCL2)-associated X protein (BAX) and decreased BCL-2 and B-cell lymphoma-extra large (BCL-xL) levels. Furthermore, pathway analysis indicated that ABL1 depletion inhibited transforming growth factor beta (TGF-β1) signaling through the insulin receptor substrate 1 (IRS1)/phosphoinositide 3-kinase (PI3K)/protein kinase B (AKT) pathway. These findings suggest that ABL1 may contribute to CRC cell proliferation and survival through the regulation of these signaling networks in the cellular models examined, although the relevance of this mechanism may vary across CRC subtypes and biological contexts. The authors therefore proposed ABL1 as a potential molecular target for CRC therapy [21]. However, evidence from an experimental model of colitis-associated cancer (CAC) indicates that ABL1 may exert an opposing, tumor-suppressive function in intestinal stem cells. Conditional deletion of ABL1 in leucine-rich repeat-containing G-protein-coupled receptor 5 (LGR5)-positive intestinal stem cells increased the number and size of azoxymethane (AOM)/dextran sulfate sodium (DSS)-induced colorectal tumors and was accompanied by enhanced proliferation and reduced apoptosis. Importantly, ABL1 deficiency impaired the response to DNA damage by reducing tumor protein p53 (TP53) phosphorylation and expression, as well as cyclin-dependent kinase inhibitor 1A (P21) and cleaved caspase-3 levels, whereas activation of upstream DNA damage response components, including ataxia-telangiectasia mutated (ATM), H2A.X histone family member (H2AX), checkpoint kinase (CHK1), and checkpoint kinase 2 (CHK2), remained largely unchanged. These findings suggest that ABL1 contributes to the activation of TP53-dependent cell-cycle arrest and apoptosis following genotoxic stress and consequently can function as a tumor suppressor during CAC development [22]. Therefore, although inhibition of ABL1 by imatinib may be therapeutically relevant in CRC models in which ABL1 functions as a pro-proliferative or pro-survival kinase, the available evidence does not support considering ABL1 an unequivocal oncogenic driver across all CRC contexts. Instead, the consequences of ABL1 inhibition may depend on the cellular context, genetic background, tumor stage, and mechanism of carcinogenesis.

### 4.2. c-KIT

c-KIT (KIT/CD117) is another established molecular target of imatinib that may have relevance in a subset of CRC. KIT is a receptor tyrosine kinase activated by stem cell factor (SCF), and early studies demonstrated that a proportion of CRC cells express both KIT and SCF, suggesting the existence of an autocrine/paracrine signaling loop. In particular, KIT and SCF were detected in two of five examined colon carcinoma cell lines, and KIT expression was confirmed at the protein level, supporting a potential role for KIT signaling in the proliferation of selected CRC cells [23]. Subsequent studies provided functional evidence for this hypothesis, demonstrating that SCF stimulation increased anchorage-dependent and anchorage-independent growth of KIT-positive CRC cells, whereas TGF-β1 suppressed KIT expression and inhibited KIT-dependent proliferation [24]. Furthermore, aberrant KIT activation was associated with several malignant phenotypes in CRC cells. In DLD-1 cells, SCF promoted survival under growth factor-deprived conditions, enhanced migration and invasion through Matrigel, and increased matrix metalloproteinase-9 (MMP9) activity, suggesting that KIT signaling may contribute not only to tumor cell proliferation but also to survival and invasive behavior [25]. Consistent with these observations, SCF/KIT signaling was subsequently shown to stimulate proliferation and invasion of KIT-positive CRC cells predominantly through the PI3K/AKT pathway, while activation of extracellular signal-regulated kinase 1 (ERK1) was associated particularly with invasive behavior [26]. Evidence from an experimental model of colorectal mucinous adenocarcinoma further supported a pro-tumorigenic function of KIT signaling, with KIT activation promoting tumor-cell proliferation through an AKT-dependent decrease in TP53 and increase in cyclin D1, while enhancing invasiveness through the ETS variant transcription factor 4 (ETV4)/matrix metalloproteinase 7 (MMP7) axis and ERK-dependent epithelial–mesenchymal transition (EMT) [27]. Nevertheless, the potential therapeutic relevance of KIT in conventional CRC is limited by the relatively low frequency and heterogeneous pattern of KIT expression. In a study of 126 colorectal carcinomas, strong KIT immunoreactivity was detected in only 1.6% of tumors, predominantly in poorly differentiated carcinomas arising at the anorectal junction [28]. Similarly, analysis of paired normal and neoplastic colorectal tissues showed KIT protein expression in only 10% of CRC cases, with most colorectal carcinomas lacking detectable KIT expression in neoplastic epithelial cells [29]. Other immunohistochemical studies reported weak-to-intermediate KIT expression in 15.2% of colorectal adenocarcinomas and strong expression in only 1.9%, with no significant association with tumor stage [30], while another series identified KIT expression in 14 of 83 colorectal cancers (17%), although expression was generally infrequent and showed no significant prognostic association [31]. Moreover, aberrant KIT expression in mucinous and non-mucinous CRC was observed in only 8% of 150 tumors, with strong expression in 4%, and was not associated with disease-free or overall survival [32]. Importantly, the molecular context also differs substantially from KIT-driven GISTs. In colorectal neuroendocrine carcinomas, for example, strong KIT expression was detected in 23% of tumors, but KIT-positive tumors lacked the exon 11 mutations characteristic of many KIT-driven GISTs, suggesting that KIT overexpression in these colorectal malignancies does not necessarily reflect an activating *KIT* mutation [33]. This distinction is particularly relevant to imatinib, because although the drug is highly effective against specific activating *KIT* mutations in GIST, experimental evidence indicates substantially weaker activity against wild-type *KIT*. Indeed, studies investigating KIT-targeted approaches in poorly differentiated CRC have noted that imatinib has limited potential against wild-type *KIT*, prompting investigation of alternative strategies for targeting KIT-overexpressing CRC cells [34]. Collectively, these findings suggest that KIT represents a biologically relevant but uncommon molecular target in CRC, with the strongest rationale for imatinib-based targeting potentially restricted to selected tumors characterized by demonstrable KIT expression and, ideally, activating KIT alterations. Thus, unlike in GIST, where KIT inhibition constitutes a central therapeutic strategy, the available CRC evidence does not currently support routine use of imatinib solely on the basis of KIT expression. Rather, KIT should be regarded as a potential target in molecularly selected CRC subgroups.

### 4.3. PDGFR

Platelet-derived growth factor receptors (PDGFRs), comprising the two receptor tyrosine kinases PDGFR-α and PDGFR-β, have emerged as potentially relevant therapeutic targets in CRC, although their biological significance differs substantially between tumor cells and the tumor-associated stroma. Early investigations demonstrated expression of platelet-derived growth factor receptor alpha (*PDGF-α*), platelet-derived growth factor receptor beta (*PDGF-β*) and *PDGFR* transcripts in human colorectal carcinomas, with *PDGFR* messenger RNA (mRNA) detected in approximately two-thirds of analyzed tumors, although *PDGFR* expression was also observed in normal tissues [35]. Subsequent immunohistochemical studies provided evidence that PDGFR-β is predominantly a stromal receptor rather than a tumor-cell receptor. In a series of 210 colorectal cancers, PDGFR-β-expressing cells were consistently detected within the tumor stroma, particularly in vascular-associated cells, whereas the tumor epithelium was invariably negative. PDGF-AB/BB-producing cells were also identified in the stroma, supporting the presence of a local PDGF signaling network involved in stromal activation [36]. Consistent with these observations, studies of human colon carcinomas demonstrated that tumor cells frequently produce PDGF-A and PDGF-B but lack corresponding *PDGFR-α/β* expression, whereas *PDGFR-β* is predominantly expressed by tumor-associated stromal cells and vascular pericytes. Importantly, stromal *PDGFR-β* expression was increased in advanced-stage tumors and was particularly pronounced in highly metastatic tumors, suggesting that PDGFR-β signaling may contribute to the establishment of a pro-metastatic tumor microenvironment rather than primarily driving proliferation of malignant epithelial cells [37].

The functional interaction between tumor cells and PDGFR-positive stromal cells provides an important rationale for considering PDGFR inhibition as a mechanism of action of imatinib in CRC. Tumor-derived PDGF-αβ/ββ was shown to activate PDGFR-β on fibroblasts, with direct tumor cell–fibroblast co-culture producing considerably stronger and more sustained PDGFR-β activation than exposure to tumor-cell conditioned medium alone. These findings indicate that cell–cell interactions within the tumor microenvironment can facilitate PDGF signaling and support a paracrine mechanism through which CRC cells modulate the phenotype and responsiveness of stromal cells [38]. Furthermore, PDGFR-β expression was found to be restricted to stromal cells in both surgical CRC specimens and orthotopic colon cancer models, emphasizing that the therapeutic relevance of this receptor cannot necessarily be evaluated by analyzing tumor-cell expression alone [39]. This distinction is particularly important for imatinib, because inhibition of PDGFR signaling may exert antitumor effects indirectly by disrupting tumor–stroma interactions, rather than through direct inhibition of CRC-cell proliferation.

Additional evidence supports a relationship between PDGFR signaling and aggressive CRC phenotypes. In a cohort of 99 colorectal carcinomas, PDGFR-α was detected in 83% and PDGFR-β in 60% of tumors, although the cellular localization differed markedly: PDGFR-α was detected in tumor cells as well as stromal pericytes, whereas PDGFR-β was restricted predominantly to stromal pericytes. PDGFR-α expression was significantly associated with lymph-node metastasis and advanced stage according to the Union for International Cancer Control (UICC) classification, while combined PDGFR-α/β expression was associated with lymphatic dissemination and advanced disease. These observations led the authors to propose PDGFR-α/β inhibition as a potential component of combination treatment strategies in CRC [40]. Moreover, *PDGFR-β* was identified among genes whose expression increased during the transition from colorectal adenoma to carcinoma, together with biological programs associated with proliferation, invasion, stromal activation and angiogenesis, suggesting that PDGFR-β may participate in the acquisition of a tumor-supportive stromal phenotype during malignant progression [41]. The clinical relevance of PDGFR signaling may also be influenced by molecular characteristics of individual tumors. In 93 CRC specimens, PDGFR-α expression was associated with *KRAS* proto-oncogene (*KRAS*) mutations, raising the possibility that PDGFR signaling may interact with established oncogenic pathways and potentially contribute to therapeutic resistance. However, whether PDGFR expression can identify patients who benefit preferentially from tyrosine kinase inhibition remains unresolved [42].

Importantly, the available data suggest that PDGFR signaling is not restricted to canonical receptor activation. A study of PDGFR-α in 103 colorectal adenocarcinomas found *PDGFR-α* overexpression in 45% of tumors, with overexpression significantly associated with exon 18 alterations, rat sarcoma (RAS) wild-type status and larger tumor diameter. These findings are of particular interest because *PDGFR-α* overexpression was associated with *RAS* wild-type CRC, a population in which resistance to anti-epidermal growth factor receptor (EGFR) therapy may occur despite the absence of canonical *RAS* mutations. The authors therefore proposed PDGFR-α as a potential therapeutic or adjunctive target in this subgroup [43]. However, the identified *PDGFR-α* alterations should not automatically be interpreted as established activating driver mutations equivalent to those observed in GIST, and their functional and predictive significance in CRC remains to be determined. Similarly, sequencing of *PDGFR-α* and *PDGFR-β* tyrosine kinase domains in CRC identified several single-nucleotide polymorphisms rather than a clear pattern of recurrent activating mutations. Of particular interest, the *PDGFR-β* exon 19 c.2601A>G variant was detected in 58% of tumors and was associated with increased PDGFR/phosphorylated (pPDGFR) protein levels and poorer survival, although its ability to predict response to PDGFR-targeted therapy remains unknown [44].

Direct experimental evidence provides stronger support for PDGFR as a pharmacological target of imatinib in CRC. In orthotopic models of colon cancer, cancer-associated fibroblast (CAFs) expressed high levels of PDGFR, and treatment with imatinib targeting PDGFR signaling inhibited the tumor-promoting stromal response. This effect was particularly relevant to bone marrow-derived mesenchymal stem cells (MSCs), which migrated into the tumor microenvironment and differentiated into CAF-like cells. Co-implantation of MSCs with KM12SM colon cancer cells enhanced tumor growth, liver metastasis, proliferation and angiogenesis while reducing tumor-cell apoptosis. Imatinib treatment counteracted these effects, prevented the increase in primary tumor and liver metastatic burden, prolonged survival, impaired MSC migration into the tumor stroma and reduced MSC survival within the tumor microenvironment. These findings provide evidence that imatinib can suppress CRC progression through a stroma-directed mechanism involving PDGFR signaling, rather than solely through direct effects on malignant epithelial cells [45]. Subsequent studies similarly reported that PDGFRβ-positive CAFs and MSC-derived stromal cells constitute important components of the tumor-promoting microenvironment and that pharmacological PDGFR inhibition can attenuate stromal activation and tumor progression [46].

The cellular consequences of PDGFR inhibition in CRC also appear to involve downstream PI3K/AKT signaling. In SW480, DLD-1 and Caco-2 CRC cells exhibiting relatively high PDGFRβ expression, genetic depletion of PDGFRβ produced moderate inhibition of proliferation accompanied by reduced PI3K pathway activation. However, pharmacological inhibition with Ki11502 (a selective inhibitor of KIT) produced stronger effects in some settings because the compound additionally inhibited other tyrosine kinases, particularly SRC. These findings are important when interpreting pharmacological studies of multi-target kinase inhibitors, as they demonstrate that the biological consequences attributed to PDGFR inhibition may partly reflect simultaneous suppression of cooperating signaling pathways. Indeed, the authors concluded that PDGFR-β inhibition alone produced only moderate effects, whereas combined inhibition of PDGFR-β, c-KIT and SRC produced a more pronounced antiproliferative response [47]. Thus, PDGFR signaling appears to function within a broader receptor tyrosine kinase (RTK) network rather than as an isolated dependency in conventional CRC cells.

The importance of the tumor microenvironment is further illustrated by studies in which PDGF signaling was pharmacologically targeted in combination with other pathways. In orthotopic and hepatic metastasis models of colon cancer, inhibition of PDGF receptor signaling with nilotinib reduced the stromal reaction, whereas mechanistic target of rapamycin (mTOR) inhibition with everolimus primarily reduced tumor-cell proliferation and microvessel density. Combined PDGF-R and mTOR inhibition produced complementary effects, including reduced stromal activation and tumor-cell proliferation, increased apoptosis and marked suppression of tumor growth at both primary and metastatic sites [48]. Although these experiments employed nilotinib rather than imatinib, they provide mechanistic support for the concept that PDGFR inhibition may be particularly effective when combined with inhibition of intracellular pathways sustaining CRC-cell growth.

The rationale for investigating imatinib in CMS4 CRC is primarily biological and preclinical. CMS4 tumors are characterized by mesenchymal and stromal gene-expression programs and are associated with an unfavorable prognosis. Preclinical studies in orthotopic colon cancer models have suggested that imatinib can inhibit PDGFR-associated stromal signaling and interfere with the tumor-promoting effects of bone marrow-derived mesenchymal stem cells within the tumor microenvironment [45]. These findings provide a biological rationale for investigating imatinib in stroma-rich, mesenchymal CRC, but do not establish that CMS4 status predicts sensitivity to the drug.

This rationale led to the Imatinib Treatment of Poor Prognosis Mesenchymal-Type Primary Colon Cancer (ImPACCT) proof-of-concept study, which selected patients with stage I–III primary CMS4 colon cancer for preoperative treatment with imatinib at 400 mg/day for 14 days before surgery [49]. The published results of this study provide preliminary human pharmacodynamic evidence, but do not establish clinical efficacy or validate CMS4 as a predictive biomarker. The findings and their limitations are discussed in Section 4.5 [50].

Collectively, the available evidence indicates that PDGFR should be regarded as a biologically relevant but context-dependent molecular target of imatinib in CRC, with the strongest rationale arising from its role in the tumor microenvironment rather than from a ubiquitous dependence of CRC epithelial cells on PDGFR signaling. PDGFR-α appears capable of being expressed by malignant epithelial cells and may be associated with advanced disease, *RAS*-wild-type status and selected genetic alterations, whereas PDGFR-β is more consistently associated with CAFs, pericytes and other stromal populations. Consequently, imatinib-mediated PDGFR inhibition may be particularly relevant in CRCs characterized by abundant activated stroma, mesenchymal/CMS4 features or a PDGFR-positive tumor microenvironment. At the same time, the heterogeneity of PDGFR expression, the relatively modest effects of selective PDGFRβ suppression in some CRC cell models, the absence of a clearly established recurrent activating *PDGFR-α*/*PDGFR-β* mutation analogous to KIT-driven GIST, and the polypharmacological nature of imatinib necessitate caution in attributing its antitumor effects exclusively to PDGFR inhibition. These considerations support further evaluation of PDGFR-directed therapy in molecularly and microenvironmentally selected CRC populations, preferably in combination with agents targeting complementary oncogenic pathways.

### 4.4. DDR1

DDR1 is a collagen-activated receptor tyrosine kinase that represents a mechanistically distinct target of imatinib compared with its canonical targets ABL1, KIT and PDGFR. DDR1 belongs to the discoidin domain receptor family, which comprises DDR1 and DDR2, and is activated by several types of extracellular matrix (ECM) collagen. Through its ability to transduce signals originating from the collagen-rich extracellular environment, DDR1 regulates cellular processes including proliferation, differentiation, adhesion, migration and invasion. DDR1 is predominantly expressed in epithelial cells and has been implicated in tumor progression in several malignancies. Importantly, chemical proteomic studies identified DDR1 as an previously unanticipated target of imatinib, establishing a biochemical basis for considering DDR1 inhibition as one component of the pharmacological activity of this multi-targeted kinase inhibitor [51]. Structural and pharmacological studies subsequently confirmed that imatinib, together with other type II kinase inhibitors such as nilotinib and dasatinib, can inhibit DDR1 kinase activity. However, because these drugs simultaneously inhibit multiple other kinases, their effects cannot be readily attributed to DDR1 alone, highlighting the importance of using selective DDR1 inhibitors or genetic approaches when evaluating DDR1-dependent phenotypes [51].

The potential relevance of DDR1 to CRC is supported by evidence demonstrating its increased expression and association with aggressive disease. In CRC specimens and cell models, increased DDR1 expression was associated with reduced expression of microRNA (miR)-199a-5p. Genetic silencing of *DDR1* or restoration of miR-199a-5p reduced colony formation, migration and invasion in LOVO and LOVE1 cells. Conversely, DDR1 upregulation was associated with increased expression of MMP2, N-cadherin and vimentin and reduced E-cadherin expression, indicating that DDR1 contributes to an EMT-like phenotype and enhanced invasive capacity [52]. These findings were complemented by clinical observations showing that DDR1 expression increases progressively from non-neoplastic colorectal tissue through adenoma to carcinoma and is associated with advanced stage, higher histological grade, lymph-node invasion and shorter overall survival. A significant relationship between DDR1 and β-catenin expression was also reported, further supporting the association between DDR1 activity and aggressive CRC biology [53]. Similarly, analysis of 126 CRC specimens demonstrated that high DDR1 expression was associated with poor differentiation and advanced tumor–node–metastasis (TNM) stage, while functional experiments in LoVo cells indicated that DDR1 promotes proliferation and metabolic reprogramming, including increased mitochondrial function, extracellular acidification and lactate production [54].

A particularly important aspect of DDR1 biology in CRC is its ability to connect tumor cells with the collagen-rich ECM. Unlike many classical growth factor receptors, DDR1 is activated directly by extracellular matrix components, allowing changes in matrix composition and organization to influence intracellular signaling. Functional studies in human colon carcinoma HT29 cells demonstrated that DDR1 activation by collagen I promotes several prometastatic phenotypes. Pharmacological inhibition or genetic silencing of DDR1 reduced collagen-dependent adhesion, proliferation, chemotactic responses and MMP-dependent invasion, accompanied by decreased AKT and ERK phosphorylation, reduced intercellular adhesion molecule 1 (ICAM1) and vascular cell adhesion molecule 1 (VCAM1) expression, lower marker of proliferation Ki-67 (MKI-67) expression, and diminished MMP9 secretion [17]. These observations indicate that DDR1 is not simply associated with an aggressive CRC phenotype but participates functionally in the cellular response to collagen and the acquisition of invasive properties.

The relationship between DDR1 and metastatic dissemination is particularly evident in models of colorectal liver metastasis. DDR1 has been identified as a RAS-independent inducer of CRC invasion and metastasis, with its kinase activity promoting BCR phosphorylation at Tyr177. This signaling event maintains β-catenin transcriptional activity required for tumor-cell invasion. Pharmacological inhibition of DDR1 with nilotinib strongly reduced CRC cell invasion in vitro and metastatic potential in intrasplenic mouse models, while also suppressing invasion of patient-derived metastatic and circulating CRC cell lines [55]. Although these experiments were performed using nilotinib rather than imatinib, they are mechanistically relevant to the proposed role of DDR1 as an imatinib target because both compounds inhibit DDR1. Nevertheless, the use of nilotinib cannot be interpreted as direct evidence that imatinib inhibits CRC metastasis through DDR1, particularly because nilotinib has a broader kinase-inhibitory profile.

Further evidence indicates that DDR1 contributes to the interaction between CRC cells and the hepatic tumor microenvironment. Tumor-cell DDR1 was shown to be a key component of the desmoplastic response surrounding colorectal liver metastases. Soluble factors originating from hepatic sinusoidal cells stimulated DDR1 activation in tumor cells, while DDR1 promoted extracellular matrix remodeling through interactions with hepatic stromal cells, including liver sinusoidal endothelial cells and hepatic stellate cells. Genetic deficiency of tumor-cell DDR1 reduced liver metastasis and impaired the associated stromal reaction, providing evidence that DDR1-mediated communication between metastatic tumor cells and the hepatic microenvironment contributes to metastatic colonization [56]. This finding is highly relevant to the potential therapeutic role of DDR1 inhibition because it suggests that inhibition of this receptor may affect not only intrinsic tumor-cell signaling but also the reciprocal interactions that allow CRC cells to adapt to the metastatic microenvironment.

The pro-invasive function of DDR1 has been further supported by studies examining collagen-rich three-dimensional environments. DDR1 overexpression in relatively low-invasive HT29 cells increased collective spheroid migration on collagen, whereas pharmacological inhibition or genetic silencing of DDR1 in highly invasive HCT116 cells markedly reduced two- and three-dimensional migration and invasion through fibrillar collagen matrices. DDR1 inhibition restored epithelial characteristics, including increased membrane localization of E-cadherin, reduced N-cadherin, and decreased membrane-type 1 matrix metalloproteinase (MT1-MMP) expression. Interestingly, phosphoproteomic analyses suggested that these effects can occur through a non-canonical signaling network involving glycogen synthase kinase-3 (GSK-3), WNK lysine-deficient protein kinase 1 (WNK1) and ribosomal S6 kinase (RSK) rather than being completely dependent on AKT or Wingless-related integration site (WNT)/β-catenin signaling. Thus the findings obtained in these experimental models suggest that DDR1 may contribute to invasive and metastasis-associated phenotypes through the regulation of cell adhesion and localized collagen remodeling [57]. These findings further strengthen the concept that DDR1 inhibition could be particularly relevant for limiting the invasive and metastatic rather than merely proliferative properties of CRC cells.

Several studies also suggest that DDR1 contributes to CRC progression through regulatory networks controlling its expression. Loss of miR-199a-5p increases DDR1 expression and promotes EMT-associated invasion and migration [52], whereas circular RNA (circRNA) derived from DNA-dependent protein kinase catalytic subunit (DNA-PKcs), circ-PRKDC, was reported to promote CRC progression through a circ-PRKDC/miR-198/DDR1 regulatory axis. circ-PRKDC and DDR1 were increased in CRC tissues and cells, while miR-198 was decreased. Depletion of circ-PRKDC inhibited proliferation, migration and invasion and promoted apoptosis and cell-cycle arrest in SW480 and HCT116 cells. miR-198 overexpression similarly inhibited CRC progression by targeting DDR1, and circ-PRKDC depletion suppressed tumor growth in vivo [58]. Another metastasis-associated regulatory pathway involves circ-nuclear receptor binding SET domain protein 2 (NSD2), which promotes CRC metastasis by sponging miR-199b-5p and thereby activating DDR1 and jagged canonical Notch ligand 1 (JAG1) signaling. Increased circ-NSD2 expression was observed in advanced and metastatic CRC, and manipulation of this pathway altered migration and metastatic behavior in vitro and in vivo, implicating DDR1 as a downstream component of a regulatory network controlling tumor–matrix interactions and metastatic dissemination [59].

The potential therapeutic importance of DDR1 extends beyond proliferation and metastasis to treatment resistance. In LoVo CRC cells, *DDR1α* overexpression increased cellular viability and reduced the cytotoxic effects of 5-fluorouracil (5-FU), whereas *DDR1α* knockdown increased apoptosis, cytochrome c release, and caspase-3 activity following 5-FU exposure. These effects were accompanied by alterations in PI3K, mouse double minute 2 homolog (MDM2), and the BAX/BCL-2 axis, suggesting that DDR1 may contribute to the cellular mechanisms underlying 5-FU resistance [60]. More recent work has further implicated DDR1 in matrix-mediated drug resistance. Collagen-rich ECM was shown to activate DDR1, which promoted collagen barrier formation, impaired immune-cell infiltration, and enhanced drug efflux, thereby contributing to matrix-mediated resistance in CRC. A multistage delivery system containing DDR1 small interfering RNA (siRNA) together with chemotherapeutic agents was capable of disrupting this DDR1-dependent matrix barrier, increasing chemotherapy penetration and facilitating immune infiltration [61]. Although these findings do not directly demonstrate that imatinib reverses 5-FU resistance through DDR1 inhibition, they provide a strong biological rationale for investigating DDR1 inhibition as a strategy for sensitizing CRC to conventional chemotherapy.

An additional and increasingly important dimension of DDR1 signaling is its influence on the tumor immune microenvironment. DDR1 was reported to be highly expressed in CRC tissues and negatively associated with patient survival. In experimental models, DDR1 promoted tumor growth predominantly in vivo and acted as a negative regulator of antitumor immunity. DDR1 reduced CD4+ and CD8+ T-cell infiltration through suppression of interleukin 18 (IL-18) synthesis and increased programmed death-ligand 1 (PD-L1) expression through c-Jun N-terminal kinase (JNK) signaling, suggesting that DDR1 can simultaneously promote tumor progression and immune evasion [62].

More recent clustered regularly interspaced short palindromic repeats (CRISPR)-based studies identified DDR1 as a potential mediator of resistance to immune checkpoint blockade in microsatellite-stable (MSS) CRC. Genetic or pharmacological DDR1 inhibition increased CD8+ T-cell infiltration and sensitized MSS CRC to programmed cell death protein 1 (PD-1) blockade. Mechanistically, collagen-bound DDR1 promoted enhancer of zeste homolog 2 (EZH2) activation and increased trimethylated histone H3 lysine 27 (H3K27me3) at the C-X-C motif chemokine ligand 10 (CXCL10) promoter, suppressing CXCL10 transcription and thereby limiting recruitment of cytotoxic lymphocytes. DDR1 expression was positively correlated with collagen I expression in MSS CRC specimens, providing a mechanistic connection between the collagen-rich ECM and immune exclusion [63]. In another study, intestine-specific *DDR1* deletion suppressed tumorigenesis in AOM/DSS and adenomatous polyposis coli multiple intestinal neoplasia heterozygous (APC^-/+^) models, reduced M2-like tumor-associated macrophages and increased CD8+ T-cell infiltration. DDR1 was shown to activate a DEAD-box helicase 21 (DDX21)–c-Jun–IL-33 pathway that promoted M2-like macrophage polarization, while DDR1 depletion enhanced the efficacy of anti-PD-1 therapy in vivo [64]. Collectively, these findings suggest that DDR1 may represent a particularly interesting target for combination strategies involving chemotherapy and immunotherapy.

Despite this extensive biological rationale, caution is required when translating DDR1 biology into the specific context of imatinib therapy in CRC. The identification of DDR1 as an imatinib target establishes that imatinib can interact pharmacologically with this receptor, but the CRC literature provides substantially stronger evidence for DDR1 as a disease-relevant target than for DDR1 as the specific mediator of imatinib-induced antitumor activity. Importantly, highly selective DDR1 inhibitors have demonstrated that DDR1 inhibition does not necessarily produce strong antiproliferative effects. In a systematic pharmacological characterization, the selective DDR1 inhibitor DDR1-IN-1 displayed limited effects on proliferation despite effective inhibition of DDR1, whereas a less selective compound with activity against additional kinases produced substantially stronger antiproliferative effects. The authors therefore proposed that DDR1 signaling may cooperate with other kinases, including SRC-family kinases, and that effective DDR1-directed treatment may require simultaneous targeting of complementary pathways such as PI3K or mTOR [51]. This observation is particularly important for interpreting imatinib studies because the drug simultaneously inhibits ABL1/ABL2, KIT, PDGFR and DDR1, making it difficult to assign a particular CRC phenotype to DDR1 inhibition alone.

Overall, DDR1 should therefore be considered an emerging and potentially important non-canonical molecular target of imatinib in CRC, but not yet an established predictive biomarker for imatinib therapy. The available evidence strongly supports a tumor-promoting role for DDR1 in CRC, particularly in collagen-dependent invasion, metastatic dissemination, tumor–stroma interactions, metabolic adaptation, chemoresistance and immune suppression. Importantly, DDR1 appears to function at the interface between the malignant cell and its extracellular environment, providing a mechanistic explanation for why its inhibition may preferentially affect invasion, metastasis and treatment resistance rather than producing profound direct cytotoxicity. The demonstration that imatinib inhibits DDR1 provides a plausible mechanism by which this receptor could contribute to the broader biological effects of imatinib. However, direct evidence demonstrating that imatinib suppresses CRC through DDR1 inhibition remains limited. Consequently, future studies should incorporate selective DDR1 inhibitors, genetic *DDR1* depletion and pharmacological comparison with imatinib to determine the relative contribution of DDR1 versus ABL, KIT, PDGFR and other kinase targets to the antitumor effects of imatinib in molecularly defined CRC populations.

### 4.5. LCK

LCK is a member of the SRC-family of non-receptor tyrosine kinases and is classically associated with T-cell receptor signaling and lymphocyte activation. Although LCK was initially considered to be restricted largely to hematopoietic cells, its expression has also been documented in non-lymphoid malignancies, including CRC. One of the earliest studies demonstrating this phenomenon identified the 56 kDa LCK protein and corresponding LCK transcripts in human colon carcinoma cell lines, showing that LCK expression was particularly pronounced in cell lines derived from metastatic tumors compared with those originating from primary tumors. These observations suggested that aberrant LCK expression may participate in the progression of a subset of epithelial malignancies, including CRC [65]. Subsequent analysis of CRC-derived cell lines demonstrated that epithelial tumor cells express alternatively spliced LCK transcripts originating from the proximal promoter, with the type 1B transcript predominating. The authors interpreted this ectopic expression as evidence of an aberrant lymphoid activation/differentiation program occurring in colorectal epithelial tumors [66].

Importantly, *LCK* expression has also been demonstrated directly in primary colorectal tumors. Analysis of 18 primary CRC specimens and two hepatic metastases showed only trace *LCK* transcript levels in normal colorectal tissues, whereas increased expression was observed in a substantial proportion of tumors. In three primary tumors, *LCK* expression was increased approximately 10- to 60-fold relative to adjacent normal tissue, while seven additional tumors exhibited more moderate increases. Moreover, one hepatic metastasis expressed higher levels of *LCK* than the corresponding primary lesion, further supporting a potential association between *LCK* expression and CRC progression or metastatic dissemination [67]. Together with the earlier cell-line observations, these findings indicate that LCK is not exclusively a lymphocyte-associated kinase in the context of CRC and can be aberrantly expressed by colorectal carcinoma cells. Nevertheless, the relatively heterogeneous expression pattern indicates that LCK should not be regarded as a universal molecular feature of CRC.

Further evidence supporting the potential clinical relevance of LCK comes from functional proteomic profiling of CRC. Analysis of 263 CRC specimens from the MD Anderson Cancer Center and validation in 462 The Cancer Genome Atlas (TCGA) CRC samples identified distinct proteomic subtypes characterized by differences in EMT and AKT/tuberous sclerosis complex (TSC)/mTOR signaling. LCK was among eight proteins whose expression was independently associated with tumor recurrence in multivariable analysis, together with proteins involved in EMT, PI3K/AKT/mTOR signaling and DNA repair. Thus, LCK may represent a component of a broader signaling phenotype associated with CRC aggressiveness and recurrence rather than acting as an isolated oncogenic driver [68]. Transcriptomic analysis of colorectal adenocarcinoma specimens has additionally identified *LCK* among genes differentially expressed in early-stage disease, together with granulysin (*GNLY*), solute carrier family 6 member 6 (*SLC6A6*) and lysosomal-associated membrane protein 2 (*LAMP2*), and proposed *LCK* as a potential biomarker for early colorectal adenocarcinoma [69]. However, these observations are primarily biomarker-level evidence and do not establish that LCK activity is required for CRC cell survival or proliferation.

The potential relevance of LCK to imatinib therapy derives from the broader kinase-inhibitory profile of imatinib. In addition to its canonical inhibition of ABL1/ABL2, KIT, and PDGF receptors, imatinib can interact with additional tyrosine kinases, including members of the SRC-family kinase group. Consequently, LCK represents a plausible noncanonical molecular target through which imatinib could influence signaling in selected CRC contexts. However, the CRC literature summarized here does not provide direct evidence demonstrating that the antitumor effects of imatinib in colorectal carcinoma are mediated specifically through LCK inhibition. In particular, the available studies demonstrate LCK expression and associations with tumor phenotype or recurrence, but do not establish a causal relationship between LCK inhibition and imatinib-induced CRC cell death. Therefore, LCK should currently be considered a potential pharmacological target of imatinib rather than an established mediator of its activity in CRC.

An additional complication in interpreting LCK expression in CRC arises from the physiological role of this kinase in immune cells. Because LCK is a central component of T-cell receptor signaling, LCK detected in bulk CRC specimens may originate from infiltrating lymphocytes rather than malignant epithelial cells. This issue is particularly important when interpreting transcriptomic and proteomic studies of whole tumor tissues, in which tumor-cell and immune-cell signals are not necessarily distinguishable. Indeed, the study identifying LCK as a recurrence-associated protein used bulk tumor proteomic analysis, and the early-stage transcriptomic study similarly analyzed tissue specimens rather than definitively establishing the cellular origin of LCK expression [68,69]. Consequently, elevated LCK in a tumor specimen should not automatically be interpreted as evidence of LCK activation within the malignant compartment. Conversely, the detection of LCK transcripts and protein in established CRC cell lines provides evidence that ectopic tumor-cell expression can occur and supports the possibility of a direct role in selected CRCs [65,66].

The immunological function of LCK further complicates the therapeutic interpretation of its inhibition. LCK is indispensable for signaling downstream of the T-cell receptor and therefore contributes to T-cell activation. Experimental targeting of LCK-related antigenic pathways has demonstrated that LCK can also participate in the tumor–immune interface. In a murine tumor model, an antibody directed against an LCK-derived cytotoxic T-lymphocyte epitope induced dendritic-cell maturation and inhibited tumor growth, while being associated with suppression of tumor-infiltrating T cells, including regulatory T cells [70]. Although this study does not investigate imatinib or CRC specifically and therefore cannot establish a therapeutic mechanism for imatinib, it illustrates the complex relationship between LCK signaling and antitumor immunity. Pharmacological inhibition of LCK could theoretically influence both malignant-cell signaling and immune-cell function, making the net consequence dependent on the cellular compartment targeted and the immunological context of the tumor.

Beyond its general role in T-cell receptor signaling, the immunological effects of imatinib may depend on drug exposure and the T-cell subset affected. Preclinical studies have shown that imatinib can induce apoptosis in effector regulatory T cells (Tregs) under specific experimental conditions while promoting tumor antigen-specific CD8+ T-cell responses [20]. These findings suggest a potential mechanism by which imatinib could alleviate Treg-mediated immunosuppression. However, the effects appear to be dose-dependent, as higher imatinib concentrations may also suppress conventional effector T-cell responses. This distinction is particularly relevant to CRC immunotherapy, given the established role of immune checkpoint inhibitors in MSI-H/dMMR disease and the limited activity of immune checkpoint inhibitor monotherapy in most MSS/pMMR tumors [71]. Nevertheless, these preclinical observations do not establish that imatinib enhances immune checkpoint blockade in CRC or that the reported effects are mediated exclusively through LCK. The potential contribution of LCK inhibition to these immunological effects, and the possibility of combining imatinib with immunotherapy, therefore require further investigation in CRC-specific models and clinical studies.

Overall, the available evidence suggests that LCK represents an emerging but insufficiently validated noncanonical target of imatinib in CRC. LCK is aberrantly expressed in a subset of CRC cell lines and primary tumors, may be more highly expressed in metastatic disease, and has been identified as a proteomic predictor of CRC recurrence [65,67,68]. Nevertheless, these observations do not establish LCK as a dominant oncogenic dependency in CRC, nor do they demonstrate that imatinib exerts clinically meaningful antitumor effects through LCK inhibition. The strongest evidence currently supports LCK as a potentially relevant component of aberrant SRC-family kinase signaling in selected CRCs. Future studies should therefore determine the cellular origin of LCK expression in individual tumors and directly compare imatinib with selective LCK/SRC-family inhibitors or genetic LCK depletion. Such experiments would be necessary to establish whether LCK expression or activity can serve as a predictive biomarker for imatinib sensitivity and whether inhibition of this kinase contributes meaningfully to the therapeutic effects of imatinib in molecularly selected CRC. The molecular targets of imatinib and their relevance in CRC pathogenesis were summarized in Figure 2.

## 5. Imatinib in Colorectal Cancer

### 5.1. Preclinical Evidence

The rationale for investigating imatinib in CRC derives from the expression and functional activation of several tyrosine kinases targeted by this drug, principally c-KIT, PDGFRs, and c-ABL. Early work demonstrated that human CRC express c-KIT and that pharmacological inhibition of this receptor with imatinib can suppress multiple malignant phenotypes. In HT29, HCT8/S11 and HCT116 cells, c-KIT was detected as a ~145 kDa receptor protein, whereas stimulation with SCF promoted tumor-cell invasion. Imatinib inhibited SCF-induced invasion with a half-maximal inhibitory concentration (IC_50_) of approximately 56 nM and inhibited HT29 proliferation with an IC_50_ of approximately 6 μM, while also inducing apoptosis and reducing tumor growth in experimental models. Importantly, SCF itself displayed proangiogenic activity, suggesting that c-KIT inhibition could simultaneously interfere with tumor-cell behavior and the tumor vascular compartment [72]. These observations were reinforced by evidence that c-KIT signaling in CRC can activate PI3K/AKT and MAPK pathways. In KIT-positive CRC cells, SCF increased proliferation and invasion, with PI3K/AKT activity showing a particularly strong association with both phenotypes, whereas MAPK/ERK1 activity was more closely associated with invasion [26].

The potential relevance of c-KIT inhibition appears to be restricted to a biologically defined subset of CRC rather than the disease as a whole. In a series of 109 CRC specimens, c-KIT overexpression was detected in 15% of primary tumors and 14% of lymph-node metastases. Notably, the tumors with increased c-KIT expression did not show hotspot c-KIT mutations, and most c-KIT-positive tumors also harbored *KRAS* and/or B-Raf proto-oncogene (*BRAF*) alterations. Nevertheless, imatinib inhibited proliferation and induced apoptosis in c-KIT-expressing CRC cell lines irrespective of their *KRAS*/*BRAF* genetic profile, suggesting that c-KIT-dependent signaling may represent a therapeutically actionable phenotype even in the absence of canonical activating KIT mutations [73].

Later work provided stronger evidence for a functional KIT-positive CRC subgroup. Analysis of 137 patient-derived colon tumors and corresponding xenografts demonstrated heterogeneous KIT/KIT ligand (KITLG) expression in a subset of tumors. Genetic knockdown of *KIT* reduced proliferation in CRC cell lines and growth of xenograft tumors, while imatinib inhibited the growth of KIT-positive colon cancer organoids and xenografts. The study further suggested that KIT may operate through autocrine and/or paracrine KITLG signaling and that endogenous KIT expression was associated with increased tumorigenicity [74]. Collectively, these findings support the concept of biomarker-guided targeting of KIT rather than indiscriminate use of imatinib in unselected CRC.

In addition to c-KIT, imatinib may act through the tumor microenvironment, particularly PDGFR signaling in stromal cells and pericytes. Experimental work showed that PDGFR is highly expressed in stromal compartments of colon tumors, whereas the cancer cells themselves may express little or no PDGFR. In orthotopic and experimental liver-metastasis models, imatinib combined with irinotecan significantly inhibited tumor growth in the cecum and liver, whereas this effect was not reproduced in subcutaneous tumors with minimal stromal reaction. The combination completely inhibited lymph-node metastasis and reduced PDGFR-β phosphorylation, stromal reaction, tumor-cell proliferation, and pericyte coverage while increasing apoptosis of both tumor cells and tumor-associated stromal cells [75]. These findings are consistent with a stromal rather than exclusively cancer-cell-directed mechanism. Similarly, imatinib inhibited PDGF-induced DNA synthesis in CAFs isolated from colorectal liver metastases. At 1 μM, it abrogated PDGF-driven fibroblast DNA synthesis, while higher concentrations reduced CAF viability. However, the latter concentrations also increased IL-6 and IL-8 expression and modestly increased vascular endothelial growth factor (VEGF) and basic fibroblast growth factor (bFGF) release, highlighting the possibility that pharmacological inhibition of stromal cells may have context-dependent effects [76]. The complexity of PDGFR-directed therapy is further illustrated by findings that *PDGF-ββ* overexpression increased vascular pericyte coverage and, unexpectedly, suppressed CRC xenograft growth, while subsequent imatinib-mediated PDGFR inhibition increased tumor growth and reduced pericyte content. The authors therefore cautioned that PDGFR inhibition may be detrimental in settings in which PDGFR is not a direct tumor-cell target and where pericytes exert vessel-stabilizing or tumor-suppressive effects [77].

Several studies nevertheless support an anti-invasive and anti-metastatic activity of imatinib in CRC. In DLD-1 cells and early-passage colorectal carcinoma cultures, imatinib inhibited anchorage-independent colony formation and cell scattering, increased susceptibility to apoptosis and markedly reduced Matrigel invasion. These effects were accompanied by reduced BCL-2 expression and induction of the pro-apoptotic B-cell lymphoma-extra small (BCL-xS) isoform. Imatinib additionally acted as a chemosensitizer when combined with 5-FU [78]. In another model, imatinib inhibited proliferation of a panel of CRC cell lines with IC_50_ values ranging from 0.3 to 3 μM. It also antagonized stromal-fibroblast-mediated stimulation of HT29 cell proliferation and inhibited stromally induced membrane-type 1 (MT1)-MMP expression and pro-MMP2 activation, providing a mechanistic link between PDGFR inhibition and reduced extracellular-matrix remodeling [79]. Consistent with this observation, imatinib altered actin cytoskeletal organization in LS180 colon adenocarcinoma cells, causing actin aggregation and reduced polymerization, which correlated with a pronounced decrease in cellular migration. At concentrations of 10–50 μM, imatinib induced phosphatidylserine externalization and caspase-3 activation, supporting an apoptotic mechanism [80]. The c-ABL axis may also contribute to these effects: inhibition of c-ABL-mediated phosphorylation of the actin-regulatory protein Abelson interactor 1 (ABI1) suppressed extracellular-matrix degradation, fibronectin attachment and invasion, while *ABI1* overexpression at invasive tumor edges was associated with an infiltrative phenotype and tumor budding in human CRC samples [81].

Imatinib also appears to influence intracellular signaling pathways beyond its canonical kinase targets. In colon cancer cells, imatinib inhibited constitutive β-catenin signaling and WNT1-induced β-catenin activity and suppressed cellular proliferation, suggesting cross-talk between tyrosine phosphorylation and the WNR/β-catenin pathway [82]. In SW480 cells, imatinib induced apoptosis, increased the G_1_-cell-cycle fraction and reduced S and G_2_/M populations. These effects were associated with increased P27 and reduced hepatocyte growth factor (HGF) expression, indicating that suppression of HGF signaling and induction of cell-cycle inhibitory mechanisms may contribute to growth arrest [83]. A broader experimental CRC study further suggested that imatinib interferes with several interconnected oncogenic pathways, including PI3K/AKT/mTOR, WNT/β-catenin and MAPK signaling, while reducing inflammatory signaling involving IL-1β/tumor necrosis factor alpha (TNF-α), nuclear factor kappa B (NF-κB) and Janus kinase 3 (JAK3)/signal transducer and activator of transcription 3 (STAT3). In the same experimental framework, imatinib was associated with increased intracellular calcium, reactive oxygen species (ROS) generation, loss of mitochondrial membrane potential, cytochrome c release, and caspase-3 activation, together with suppression of angiogenic signaling, supporting a pleiotropic rather than strictly single-target mechanism [84].

The effects of imatinib may also be particularly relevant to residual disease and therapy-resistant CRC populations. In a murine CT-26 model, conventional doxorubicin treatment enriched a residual population with cancer-stem-cell-like characteristics. Subsequent imatinib treatment potently suppressed clonogenicity of doxorubicin-refractory cells under both normoxic and hypoxic conditions. Importantly, imatinib prevented tumor formation in 50% of mice injected with doxorubicin-pretreated CT-26 cells and delayed tumor formation in the remaining animals, whereas it did not prevent tumor development when administered against chemotherapy-naïve CT-26 cells. These findings suggest that imatinib may be more effective as a sequential treatment directed against therapy-resistant, stem-like residual disease than as a conventional cytoreductive monotherapy [85].

A related study in human CRC demonstrated that imatinib reduced clonogenicity of 5-FU-refractory HT29 and HCT116 cells under both normoxia and hypoxia and downregulated stemness-associated genes in cells undergoing renewal under normoxic conditions. The combination of imatinib with the vitamin-D analogue PRI-2191 produced greater suppression of stemness-related gene expression in HCT116/5-FU cells than imatinib alone, whereas another vitamin-D analogue, PRI-1906, abolished this effect. These findings support a potential role for imatinib in limiting cancer-cell plasticity and recurrence after conventional chemotherapy [86].

Additional evidence indicates that c-KIT inhibition may be relevant to specific histopathological CRC phenotypes. In models of mucinous colorectal adenocarcinoma (MCA), SCF/c-KIT signaling was strongly activated, and genetic or pharmacological inhibition of this pathway altered mucin 2 (MUC2) production and atonal bHLH transcription factor 1 (*ATOH1*) expression. In mice, loss-of-function *c-KIT* mutants developed non-mucinous rather than mucinous tumors following azoxymethane/dextran sulfate sodium exposure, whereas imatinib suppressed *ATOH1* expression in CRC cells stimulated by SCF or *c-KIT* overexpression. Mechanistically, SCF/c-KIT-mediated activation of AKT maintained inhibitory phosphorylation of glycogen synthase kinase 3 beta (GSK3β), thereby sustaining *ATOH1* and *MUC2* expression [87].

A complementary study showed that SCF/c-KIT signaling promotes mucus secretion through a protein kinase C delta–myristoylated alanine-rich protein kinase C substrate (PKCδ-MARCKS) pathway. Imatinib reduced mucus secretion in mice, while modulation of SCF/c-KIT signaling in HT29 and LS174T cells altered MARCKS activity. These findings suggest that aberrant KIT signaling may contribute not only to tumor-cell growth but also to the characteristic mucinous phenotype and secretory biology of MCA [88].

The rationale for investigating imatinib in CRC was provided in Figure 3.

### 5.2. Combination Therapies and Imatinib

Combination therapy is a particularly recurrent theme in the preclinical literature. Imatinib sensitized CRC cells to 5-FU, with particularly pronounced growth inhibition in HT29 cells [78,79]. In vivo, addition of imatinib to irinotecan substantially inhibited orthotopic colon tumors and experimental liver metastases and completely prevented lymph-node metastasis in one model [75].

A broader strategy targeting tumor-cell EGFR/vascular endothelial growth factor receptor (VEGFR) together with stromal PDGFR was also investigated using AEE788, imatinib and irinotecan. The triple combination produced the greatest inhibition of primary tumor growth and metastasis, indicating that simultaneous targeting of heterogeneous signaling compartments may be more effective than inhibition of an individual receptor pathway [89]. A vascular-normalization approach provided another rationale for combining imatinib with anti-VEGF therapy. Low-dose bevacizumab plus imatinib generated similar growth inhibition to more intensive VEGF inhibition but avoided excessive collagen-IV deposition and produced a more mature, normalized and perfused vasculature, with reduced vascular leakiness, hypoxia and a greater proportion of vessels with open lumens [90]. This mechanism is particularly important because it provides a pharmacological rationale for imatinib that does not depend exclusively on direct cytotoxicity against CRC cells.

Several additional combinations have shown activity in vitro. Selenite synergistically increased imatinib cytotoxicity in HCT116 cells, with increased caspase-3 activity, reduced *BCL-2* expression, DNA fragmentation, inhibition of cytoprotective autophagy, and S-phase arrest [91].

Celecoxib combined with imatinib produced greater loss of HT29 cell viability and increased caspase-3 activity, accompanied by reduced cyclooxygenase-2 (*COX-2*) expression, suggesting that simultaneous modulation of tyrosine-kinase and COX-2-associated pathways may enhance activity [92]. Similarly, half-dose combinations of imatinib and the non-COX-2-inhibitory celecoxib analogue 2,5-dimethyl-celecoxib produced apoptosis in HT29 cells comparable to full-dose imatinib, with increased caspase-3 activity and caspase-3 gene expression [93].

Metformin synergistically enhanced imatinib cytotoxicity in HCT15 cells and induced S/G_2_/M arrest through cyclin-B1 reduction while suppressing phosphorylated ERK. The combination also induced autophagy. Inhibition of autophagy with chloroquine further reduced cell viability, indicating that treatment-associated autophagy was predominantly cytoprotective in this model [94].

Brassinin similarly synergized with imatinib in SW480 cells, increasing cytotoxicity and apoptosis, causing G_0_/G_1_ arrest and suppressing MMP-9 activity and expression, thereby providing evidence for a potential anti-metastatic component [95].

An especially interesting strategy involves modulation of drug transport and cellular drug exposure. In HCT116 cells, thymoquinone markedly reduced imatinib IC_50_ values: the imatinib IC_50_ decreased from 105 and 72 μM at 48 and 72 h, respectively, to 7 and 5.5 μM with 10 μM thymoquinone, and to 5.6 and 4.6 μM with 20 μM thymoquinone. Combination-index and dose-reduction analyses indicated significant synergism. Thymoquinone increased intracellular imatinib accumulation and downregulated ATP-binding cassette subfamily B member 1 (*ABCB1*), ATP-binding cassette subfamily G member 2 (*ABCG2*), and human organic cation transporter 1 (*hOCT1*), providing a mechanistic explanation for enhanced intracellular drug exposure [96]. A subsequent study reported that the same combination induced both autophagy and apoptosis, with evidence from apoptosis markers, AMP-activated protein kinase (AMPK)/prostate apoptosis response protein 4 (PAR-4) signaling and colony-formation assays supporting programmed cell death [97].

More recently, imatinib combined with zebularine produced synergistic growth inhibition in HCT116 cells, enhanced oxidative stress, increased apoptosis and necrosis, induced S-phase arrest, reduced MMP-2/MMP-9 and BCL-2, and inhibited EGFR expression, although these observations remain entirely preclinical and require independent validation before translational interpretation [98].

The combined preclinical evidence therefore suggests that imatinib possesses several potentially useful activities in CRC: direct growth inhibition and apoptosis in KIT-positive cells, suppression of tumor-cell migration and invasion, interference with stromal PDGFR signaling, modulation of extracellular-matrix remodeling, impairment of therapy-resistant/stem-like residual cells, vascular normalization, and enhancement of the activity of established or experimental agents. At the same time, the evidence also demonstrates important biological limitations. *KIT* expression is heterogeneous, canonical *KIT* mutations are not required for activity, and PDGFR inhibition may have unfavorable consequences in tumors in which stromal pericytes exert tumor-suppressive functions [73,74,77].

Moreover, imatinib does not universally enhance combination therapy: notably, it reduced TNF-related apoptosis-inducing ligand (TRAIL)-induced apoptosis in HCT116 and SW480 cells, apparently by inhibiting the c-ABL/tumor protein p73 (P73)/JNK/P38 stress-signaling cascade required for TRAIL cytotoxicity [99]. Thus, preclinical literature supports imatinib primarily as a context-dependent, biomarker-informed and combination-oriented therapy, rather than as a broadly active cytotoxic agent for unselected CRC.

These observations should be interpreted alongside the predominantly positive results reported for other preclinical combinations, including imatinib with fluoropyrimidines or selected experimental compounds. Differences in genetic background, basal kinase activity, drug exposure, and the signaling pathways required for apoptosis may contribute to the divergent responses. Therefore, the efficacy of an imatinib-containing combination should not be inferred from its individual components or from synergistic effects observed in a different cellular model. Mechanistic validation and appropriate comparisons with each agent alone are necessary before these combinations can be considered suitable for clinical investigation.

### 5.3. Novel Formulations of Imatinib

The conventional systemic administration of imatinib creates an additional translational challenge: although the drug has established pharmacological activity against several tyrosine kinases, its repositioning for CRC requires sufficiently high drug exposure at tumor sites while limiting systemic toxicity and overcoming mechanisms such as multidrug resistance (MDR). The formulation studies focus mainly on increasing tumor accumulation, controlling release, circumventing drug efflux, improving cellular uptake, and enabling localized delivery. One of the earliest approaches involved hyaluronan-coated liposomes containing an imatinib–sodium deoxycholate conjugate. The imatinib conjugate was encapsulated into liposomes with a mean size of approximately 56.6 nm and an entrapment efficiency of 99.1%. Subsequent hyaluronan coating increased particle size to approximately 159 nm. The formulation was designed specifically for MDR colon cancer and showed enhanced internalization in CD44-positive HT29 and Colo-320 cells. The authors also reported increased uptake of a P-glycoprotein (P-GP) substrate in P-GP-positive Colo-320 cells and low opsonization activity, supporting the possibility that hyaluronan-mediated targeting and altered intracellular trafficking could partially overcome barriers associated with MDR [100].

Niosomal encapsulation represents another strategy intended to improve the therapeutic index of imatinib. An optimized imatinib-loaded niosomal formulation developed using a Box–Behnken design showed a vesicle size of 425.36 nm, a zeta potential of −62.4 mV and an entrapment efficiency of 82.96%. Drug release reached 89.45% after 24 h, with only 18.93% released during the initial 2 h, indicating a comparatively controlled-release profile. The optimized formulation showed substantially improved activity relative to free imatinib, with IC_50_ values 6.7-fold lower in MCF-7 cells, 16.4-fold lower in HCT116 cells and 7.3-fold lower in HepG2 cells. Importantly for translational considerations, IC_50_ values in the tested normal-cell model were 3–11 times higher than those observed in the cancer cell lines, suggesting an improved degree of cancer-cell selectivity in vitro [101]. Although not all of these findings were generated in CRC models, the markedly improved activity observed in HCT116 cells supports continued evaluation of this formulation strategy in colorectal oncology.

Chitosan-based nanoparticles have been investigated more specifically as a CRC-targeting system. An optimized chitosan polymeric nanoparticle formulation developed by ionic gelation had a particle size of approximately 208 nm, a zeta potential of −32.56 mV, 68.52% drug entrapment and 86.45% cumulative drug release. Following intravenous administration, fluorescence microscopy demonstrated enhanced nanoparticle accumulation in epithelial colon cells. The formulation displayed only 0.46% hemolysis, while histopathological examination did not identify tissue damage, supporting preliminary biocompatibility. Importantly, encapsulated imatinib produced greater and more controlled cytotoxicity than free drug in CT26 colon carcinoma cells, suggesting that nanoparticle-mediated delivery may improve exposure at the tumor site while potentially reducing systemic toxicity [102].

A subsequent chitosan nanoparticle system coated with D-α-tocopheryl polyethylene glycol 1000 succinate (TPGS) was designed to further improve cellular uptake. In HCT116 cells, CS-imatinib-TPGS nanoparticles outperformed free imatinib with respect to cytotoxicity, apoptosis induction, cellular uptake and inhibition of cell migration. The nanoparticles also showed sustained release at pH 5.5 without an initial burst, and the broader formulation evaluation included mitochondrial membrane potential, DNA fragmentation, ROS, cell-cycle analysis, hemocompatibility and platelet-aggregation assays. In vivo experiments in rats provided preliminary pharmacokinetic and acute-toxicity information [103].

Another emerging approach is the use of localized, injectable in situ-forming gels. A rosin/cinnamon-oil-based formulation was developed to provide sustained imatinib release at the site of administration. The formulations showed acceptable injectability, with viscosity below approximately 267 cP. Incorporation of cinnamon oil altered the mechanical properties of the gel and promoted higher imatinib release over a period extending to 28 days. All formulations displayed cytotoxicity against HCT116 and HT29 cells, supporting the concept of prolonged local drug exposure as an alternative to repeated systemic dosing [104].

An injectable gamboge-based in situ gel was subsequently developed with even lower viscosity, below 80 cP, and demonstrated formation of a porous structure in an aqueous environment, allowing sustained imatinib release over approximately 14 days. A 1% *w*/*w* imatinib formulation reduced HCT116 and HT29 viability to below 20% after 2 days of incubation. The authors proposed that such a localized delivery system could potentially increase intratumoral/local concentrations while reducing systemic exposure and treatment-related adverse effects [105].

Multifunctional co-delivery systems are particularly attractive because they seek not merely to modify pharmacokinetics but also to overcome specific mechanisms of therapeutic resistance. A liposomal system carrying imatinib in the internal aqueous phase and TRAIL incorporated into the membrane was developed to simultaneously address TRAIL resistance and systemic imatinib toxicity. The particles were approximately 150 nm and showed increased accumulation in TRAIL-resistant cells and in HT29 tumor-bearing mice. In vitro, the formulation increased HT29 cell killing by approximately 50%, induced apoptosis and caused accumulation of up to 32.3% of cells in M phase. In vivo, the formulation achieved 61.8% tumor suppression in a HT29 xenograft model with relatively little apparent toxicity [106]. This finding is noteworthy because conventional free-drug combination can be complicated by the observation that imatinib actually inhibits TRAIL-induced apoptosis in HCT116 and SW480 cells through suppression of the c-Abl/p73/JNK/p38 pathway [99]. The liposomal strategy therefore illustrates an important concept: drug-delivery architecture may alter the biological consequences of combining agents that can be antagonistic when administered as free drugs.

The newest formulation approach involves chitosan-coated silver nanourchins. The imatinib-loaded particles had a size of approximately 193 nm and 85.7% drug-entrapment efficiency. In HCT116 cells, the nanocarrier produced an IC_50_ of approximately 0.4 μg/mL and increased apoptosis and ROS generation relative to imatinib alone. Sustained drug release exceeded 85% over 60 h, while additional testing suggested antiangiogenic activity, downregulation of BCL2 and upregulation of apoptotic genes. Pharmacokinetic studies in Wistar rats demonstrated approximately 1.8-fold improved bioavailability, which the authors interpreted as potentially enabling higher colon exposure with reduced systemic toxicity. The platform also showed activity against selected pathogenic bacteria without apparent suppression of beneficial microbes [107].

Taken together, these formulation studies suggest that the major translational opportunity may not be simply to reproduce the pharmacology of oral imatinib, but to change its spatial and temporal distribution. Liposomes, niosomes and polymeric nanoparticles may enhance cellular internalization and partially circumvent drug-efflux mechanisms, whereas in situ-forming gels may provide prolonged local release. Co-delivery platforms can additionally integrate imatinib with agents that target complementary pathways. Nevertheless, the evidence remains predominantly exploratory: the demonstrated improvements in cytotoxicity, biodistribution or pharmacokinetics arise largely from cell culture and animal studies, and there is no evidence in the supplied material that any of these CRC-directed formulations has undergone clinical evaluation in CRC patients. Therefore, these systems should currently be regarded as preclinical strategies for overcoming the pharmacological limitations of imatinib rather than clinically validated formulations (Figure 4). Preclinical evidence supporting the therapeutic potential of imatinib in CRC was summarized in Table 1.

### 5.4. Clinical Evidence

In contrast to the substantial experimental literature, clinical evidence supporting imatinib as an active CRC treatment remains limited and is largely derived from small phase I or exploratory phase I/II studies in combination with chemotherapy, antiangiogenic therapy or EGFR-targeted therapy. Importantly, the available clinical studies do not establish imatinib monotherapy as an effective standard treatment for CRC. Rather, they primarily test whether the drug can be administered safely at pharmacologically relevant doses and whether its proposed effects on PDGFR-dependent tumor stroma and interstitial fluid pressure can be translated into improved drug delivery or therapeutic activity.

An early phase I trial evaluated imatinib in combination with 5-FU and leucovorin in patients with chemotherapy-refractory gastrointestinal malignancies, including CRC. Thirty patients were treated across five dose levels, with imatinib escalated from 300 to 700 mg/day. Treatment-related adverse events included anemia (43%), nausea (33%), fluid retention (27%), elevated γ-glutamyl transferase (20%) and diarrhea. Dose-limiting toxicities consisted of severe neutropenia, central fluid retention and severe nausea, establishing a maximum tolerated dose of 600 mg/day when administered in a week-on/week-off schedule. Pharmacokinetic analysis did not identify major differences in imatinib exposure before versus during chemotherapy. Although only a minor response was observed, some patients experienced clinical signs of activity, including resolution of ascites and improvement in performance status. Thus, the study established feasibility but provided only limited evidence of antitumor efficacy [108].

A subsequent phase I study specifically addressed advanced CRC using imatinib in combination with modified folinic acid, 5-FU, oxaliplatin (mFOLFOX6) and bevacizumab. The rationale was that inhibition of PDGFR could decrease tumor interstitial fluid pressure and thereby improve chemotherapy delivery. Ten patients were enrolled, with imatinib evaluated at 400, 600 and 800 mg/day. Dose escalation was limited by hematological toxicity: dose-limiting toxicities occurred in three of six patients receiving 600 mg/day, including two cases of febrile neutropenia and one patient with grade 3 infection and grade 4 neutropenia. Grade 3/4 neutropenia occurred in more than 60% of the overall cohort. Importantly, imatinib clearance decreased after chemotherapy, whereas the pharmacokinetics of oxaliplatin and 5-FU were not significantly altered by imatinib. The resulting maximum tolerated dose (MTD) was 400 mg/day of imatinib in combination with full-dose mFOLFOX6–bevacizumab. The study therefore demonstrated that pharmacologically relevant PDGFR inhibition could be achieved, but at the cost of substantial hematological toxicity that prevented dose escalation [109].

The largest clinical dataset in the supplied material comes from the AIO KRK 0205 phase I/II trial, which combined imatinib with capecitabine plus oxaliplatin (XELOX) and bevacizumab as first-line treatment for metastatic CRC. The therapeutic concept was based on combined PDGFRβ and VEGF inhibition, with the expectation that vascular normalization would improve delivery of chemotherapy to tumor tissue. Imatinib was administered at 300 mg/day on days 1–21 of a 21-day cycle together with capecitabine, oxaliplatin and bevacizumab. A total of 49 patients were evaluated. The lower imatinib dose level was selected for phase II because gastrointestinal toxicity at higher doses was considered clinically significant. Nevertheless, the regimen produced a 6-month progression-free survival (PFS) rate of 76%, median PFS of 10.6 months and median overall survival of 23.2 months. Hematological toxicity was generally mild, while sensory neuropathy and diarrhea were the most frequent grade 3 toxicities. The authors concluded that the combination was tolerable and demonstrated promising efficacy [110]. However, because this was a non-comparative early-phase study and imatinib was administered together with three established active anticancer agents, these outcomes cannot be interpreted as evidence that imatinib itself improved survival.

Another phase I study evaluated imatinib together with bevacizumab and metronomic cyclophosphamide in patients with refractory stage IV CRC. Thirty-five patients received treatment with bevacizumab 5 mg/kg every 2 weeks, cyclophosphamide and imatinib according to a dose-escalation design. The MTDs were 50 mg/day cyclophosphamide, 400 mg/day imatinib and 5 mg/kg bevacizumab. Dose-limiting toxicities included nausea/vomiting, neutropenia, hyponatremia, fistula and hematuria, and the toxicity observation window had to be extended to 42 days because some adverse events occurred later than expected. Seven patients (20%) achieved stable disease lasting more than 6 months. However, circulating tumor, endothelial and immune-cell subsets did not correlate with PFS, emphasizing the absence of a validated biomarker of benefit in this small study [111]. Again, prolonged disease stabilization in a minority of heavily pretreated patients supports biological activity worth investigating but does not demonstrate a therapeutic effect attributable specifically to imatinib.

A different clinical strategy attempted to identify patients for imatinib treatment according to activation of its molecular targets. In a pilot phase I/II trial, patients with metastatic CRC and biopsiable liver metastases underwent reverse-phase protein array analysis to assess phosphorylation of c-KIT, PDGFR and c-ABL. Seven patients were enrolled, with the experimental group receiving imatinib followed by panitumumab and the control group receiving panitumumab alone. Sequential pre- and post-treatment biopsies were available for three patients, and two showed decreased activation of the targeted pathways and downstream substrates following therapy. However, combination treatment was also associated with activation of the AKT/mTOR pathway and several other receptor tyrosine kinases, indicating potential compensatory signaling and adaptive resistance. Although the study did not establish efficacy, it provided an important proof of concept for pathway-activation-based patient selection and real-time assessment of target engagement [112]. This approach is particularly consistent with the preclinical evidence indicating that imatinib activity may depend on the presence and functional activity of KIT/PDGFR/c-ABL signaling rather than on CRC histology alone.

One of the most compelling clinical observations concerns imatinib’s proposed effect on the tumor microenvironment. Because high interstitial fluid pressure (IFP) can impair penetration of anticancer drugs into solid tumors, PDGFR inhibition has been proposed as a means of improving tissue perfusion and drug delivery. In a study of nine patients with progressive CRC metastases despite established therapies, oral imatinib at 400 mg/day was administered for 6–7 days and evaluated by ^15^O-water positron emission tomography/computed tomography (PET/CT). Imatinib increased the water-perfusable tissue fraction (PTF) from 0.62 ± 0.12 to 0.69 ± 0.13 (P = 0.02), without significantly altering tumor blood flow. The effect was no longer statistically detectable one week after discontinuation. In contrast, a recombinant human interleukin-1 receptor antagonist (IL-1Ra), anakinra, did not significantly alter PTF or tumor blood flow. These data constitute direct human evidence that imatinib can modify a physical property of metastatic CRC tissue relevant to drug delivery and support the proposed mechanism whereby PDGFR inhibition may improve penetration of co-administered anticancer agents [113]. Importantly, however, increased PTF is a pharmacodynamic effect rather than evidence of clinical tumor regression or survival benefit.

Taken together, the clinical evidence provides a relatively coherent picture. First, imatinib can be administered to patients with advanced or refractory CRC, including in combination with fluoropyrimidines, oxaliplatin, bevacizumab, cyclophosphamide and panitumumab, but dose intensity may be constrained by hematological and gastrointestinal toxicity [108,109,110,111]. Second, combinations involving bevacizumab and chemotherapy have produced encouraging disease-control parameters in early-phase studies, particularly the 76% 6-month PFS rate and median OS of 23.2 months reported in AIO KRK 0205 [110]. Third, imatinib produces measurable pharmacodynamic effects in human CRC metastases, including an increase in water-perfusable tumor tissue, supporting its proposed stromal/PDGFR-directed mechanism [113]. Fourth, molecular profiling may have potential value for patient selection, although the very small personalized-therapy study also demonstrated treatment-associated compensatory activation of AKT/mTOR and other receptor tyrosine kinases [112].

Despite these encouraging signals, the clinical evidence remains insufficient to conclude that imatinib is an effective standalone treatment for CRC. The available studies are small, predominantly phase I or exploratory phase I/II trials, and generally involve combination regimens that do not provide randomized evidence that adding imatinib improves clinically meaningful endpoints over contemporary standard therapy. Moreover, the toxicity observed at higher doses, particularly neutropenia, febrile neutropenia, gastrointestinal toxicity, fluid retention and other treatment-related complications, limits simple escalation of systemic exposure [108,109,110,111]. The translational value of imatinib may consequently lie less in conventional monotherapy and more in selective inhibition of KIT/PDGFR-dependent CRC, modulation of the tumor microenvironment to enhance delivery of other therapies, treatment of residual stem-like disease following cytotoxic therapy, and incorporation into rational combination or tumor-targeted delivery systems.

### 5.5. Comparative Clinical Evidence from Other Tyrosine Kinase Inhibitors

The clinical experience with other tyrosine kinase inhibitors (TKIs) provides an important comparative context for interpreting the therapeutic potential of imatinib in CRC. Regorafenib, an oral multikinase inhibitor targeting several pathways involved in tumor growth, angiogenesis, and the tumor microenvironment, demonstrated a statistically significant survival benefit in the randomized phase III CORRECT trial in patients with treatment-refractory metastatic CRC. Median overall survival was 6.4 months with regorafenib compared with 5.0 months with placebo, corresponding to a hazard ratio for death of 0.77 [114]. Although the absolute survival benefit was modest and treatment-related toxicity was substantial, this trial provided controlled clinical evidence supporting regorafenib as a therapeutic option in a defined treatment setting.

In contrast, the randomized phase III study evaluating sunitinib in combination with FOLFIRI in previously untreated metastatic CRC was discontinued following an interim analysis indicating potential futility. Median progression-free survival was 7.8 months with sunitinib plus FOLFIRI and 8.4 months with FOLFIRI alone, with no statistically significant evidence of superiority. The sunitinib-containing regimen was also associated with more severe adverse events and a less favorable safety profile [115]. These results demonstrate that inhibition of multiple receptor tyrosine kinases, including pathways associated with angiogenesis and stromal signaling, does not necessarily translate into improved outcomes when incorporated into a combination regimen.

Together, these studies illustrate the importance of clinical validation rather than extrapolation from the molecular target profile of a TKI. Regorafenib provides evidence of a modest but significant survival benefit in previously treated metastatic CRC, whereas sunitinib demonstrates that a biologically plausible multi-target strategy may fail to improve treatment outcomes and may increase toxicity. In comparison, the available evidence for imatinib remains insufficient to establish a reproducible clinical benefit or a validated biomarker-defined responsive population. Future studies should therefore prioritize mechanistically justified patient selection, pharmacodynamic confirmation, and controlled evaluation of treatment benefit rather than assuming that inhibition of shared kinase pathways will produce comparable clinical effects.

### 5.6. Future and Ongoing Clinical Studies

Clinical investigation of imatinib in CRC has remained limited and has primarily focused on biomarker-selected populations or combination strategies designed to exploit signaling pathways involving PDGFR, KIT, or other tyrosine kinases rather than on unselected CRC populations. One of the earliest dedicated studies was a phase II trial of imatinib in stage IV CRC (NCT00041340), in which patients with metastatic disease were selected on the basis of tumor expression of potential imatinib targets, including c-KIT, ABL/ARG and PDGFR. The study was designed not only to assess objective tumor response and toxicity but also to characterize the biological effects of imatinib on the c-KIT/PDGFR signaling system and downstream pathways. Patients received oral imatinib twice daily for an initial 8-week period, with continuation in patients achieving stable disease or better until disease progression or a maximum specified treatment duration. The biological rationale was based on the observation that a subset of CRCs expresses imatinib-sensitive receptor or non-receptor tyrosine kinases and that inhibition of these pathways could interfere with tumor growth. Importantly, however, the biomarker-selection strategy illustrates an early recognition that imatinib was unlikely to be effective as an indiscriminate therapy for metastatic CRC and that therapeutic activity might depend on the presence of an activated molecular target. The trial was completed as a phase II study, but it did not establish imatinib as a standard treatment for metastatic CRC. ClinicalTrials.gov: NCT00041340.

A related translational approach was subsequently evaluated in the phase I/II NITMEC study (NCT00867334), which investigated imatinib in combination with the anti-EGFR monoclonal antibody panitumumab in patients with stage IV CRC metastatic to the liver. The mechanistic rationale was based on the observation that although activating *KRAS* mutations strongly predict resistance to anti-EGFR therapy, a proportion of patients with wild-type *RAS* tumors also fail to respond to panitumumab. The investigators therefore hypothesized that activation of alternative receptor tyrosine kinase pathways, particularly PDGF/PDGFR and KIT, could provide bypass signaling and contribute to resistance to EGFR blockade. Accordingly, patients were prescreened for activation of c-KIT/PDGFR-related pathways using a proteomic assay, and the study incorporated analyses of phosphorylation levels of imatinib-sensitive targets and tumor burden. The trial used a prospective, non-randomized, two-arm design, comparing treatment involving imatinib, initially as monotherapy followed by imatinib plus panitumumab, with standard-of-care panitumumab. The study was completed in 2011 with an actual enrollment of only 10 patients, substantially limiting the ability to draw definitive conclusions regarding efficacy. Nevertheless, NCT00867334 is mechanistically important because it represents one of the earliest attempts to use functional pathway activation rather than conventional clinicopathological characteristics to identify patients potentially susceptible to imatinib-based therapy.

Another clinical strategy was to combine imatinib with established cytotoxic chemotherapy. The phase I/II study NCT00183833 evaluated oral capecitabine plus imatinib in patients with advanced solid tumors, including CRC. The rationale was predominantly pharmacological and translational: capecitabine provides systemic exposure to 5-FU, a standard component of CRC treatment, whereas imatinib was intended to inhibit signaling through its susceptible tyrosine kinase targets in tumors that had progressed after standard treatment. The principal objective was to establish the dose-limiting toxicity and MTD of imatinib administered with a fixed, CRC-relevant dose of capecitabine, while secondary endpoints included response rate, time to progression, survival and exploratory molecular correlates of efficacy. The study enrolled 40 patients overall and was completed in 2010. ClinicalTrials.gov (https://clinicaltrials.gov/) currently classifies the study as a phase I interventional trial, despite the historical title referring to phase I/II development. Thus, this study primarily provided dose-finding and safety information rather than definitive evidence of clinical efficacy of imatinib in CRC.

The potential of imatinib to modulate the tumor vasculature and microenvironment was investigated in NCT00784446, a phase I/II study evaluating first-line capecitabine/oxaliplatin (XELOX) plus bevacizumab and imatinib in patients with stage IV CRC. The biological rationale differed from studies directly targeting tumor-cell proliferation. Imatinib was incorporated primarily because of its ability to inhibit PDGFR signaling, which is important for pericyte recruitment and vascular maturation. Inhibition of PDGFR-mediated pericyte–endothelial interactions was hypothesized to destabilize tumor vasculature and thereby increase sensitivity to VEGF inhibition by bevacizumab. The regimen therefore combined simultaneous targeting of the PDGF/PDGFR and VEGF/VEGFR angiogenic axes, together with standard cytotoxic chemotherapy using capecitabine and oxaliplatin. Patients received imatinib during days 1–21, capecitabine on days 1–14, and oxaliplatin plus bevacizumab on day 1 of 22-day cycles. The study enrolled 51 patients and was completed in 2012. As a phase I/II study primarily assessing the safety and tolerability of the combination, it was not designed to provide definitive evidence that imatinib improves survival compared with standard bevacizumab-containing chemotherapy. Nevertheless, it provides an important clinical test of the concept that PDGFR inhibition could enhance anti-VEGF therapy through vascular normalization or destabilization of pericyte support.

A further attempt to exploit the PDGFR-dependent stromal component of CRC was made in the ImPACCT trial (NCT02685046), a phase II proof-of-concept study specifically targeting the aggressive CMS4 of colon cancer. CMS4 tumors are characterized by mesenchymal, stromal and stem-like gene-expression programs and are associated with an unfavorable prognosis and an increased risk of recurrence. The rationale for imatinib treatment was derived from preclinical observations suggesting that inhibition of PDGFR and KIT signaling could suppress the mesenchymal phenotype of CMS4 colon cancer. Rather than administering imatinib to an unselected CRC population, the investigators used a reverse transcription quantitative polymerase chain reaction (RT-qPCR)-based molecular assay to prospectively identify patients with CMS4 tumors. Selected patients with newly diagnosed stage I–III colon cancer were scheduled to receive imatinib 400 mg/day for two weeks before surgical resection, allowing paired pre-treatment and post-treatment tumor samples to be obtained. RNA sequencing was planned to determine whether short-term imatinib exposure induced molecular changes consistent with attenuation of the aggressive CMS4 phenotype. This design represented an important conceptual shift from conventional late-stage efficacy trials because the preoperative window allowed direct assessment of on-target biological effects within human tumor tissue before definitive surgery. However, the trial was ultimately terminated because of low accrual, preventing the study from providing definitive evidence that imatinib modifies clinical outcome in CMS4 CRC.

Published analysis of the ImPACCT proof-of-concept study subsequently reported changes in selected gene-expression markers in paired tumor samples from the five patients who received imatinib. Expression of mesenchymal-associated genes, including ZEB1, PDGFRA, PDGFRB, and CD36, decreased, whereas epithelial junction-related genes such as CDH1, CTNNA1, and JUP increased. However, the average probability of CMS4 classification did not decrease significantly, and the study was too small to establish therapeutic efficacy. These findings should be interpreted as preliminary human pharmacodynamic evidence rather than proof that CMS4 predicts benefit from imatinib [50].

Finally, another early-phase study, NCT01271166, investigated imatinib in combination with mFOLFOX6 plus bevacizumab (mFOLFOX–Avastin) in patients with advanced malignancies, using a phase I dose-finding design to establish the maximum tolerated dose of imatinib when added to a standard CRC regimen. The mechanistic rationale was consistent with the vascular-stromal hypothesis evaluated in NCT00784446: simultaneous inhibition of PDGFR signaling by imatinib and VEGF signaling by bevacizumab was intended to interfere with complementary mechanisms supporting tumor angiogenesis, while mFOLFOX provided established cytotoxic activity. The principal objective of such a phase I design was determination of dose-limiting toxicity and the recommended dose for subsequent investigation, rather than demonstration of superiority in progression-free or overall survival. Thus, this study should be interpreted primarily as a feasibility and safety investigation of incorporating imatinib into a contemporary CRC chemotherapy/antiangiogenic backbone, rather than as evidence of therapeutic efficacy.

Taken together, the ClinicalTrials.gov record indicates that clinical development of imatinib in CRC has followed several related but distinct hypotheses: direct inhibition of tumor-associated KIT/PDGFR or ABL-family signaling in molecularly selected tumors (NCT00041340), blockade of alternative growth-factor signaling to overcome resistance to EGFR inhibition (NCT00867334), combination with fluoropyrimidine-based chemotherapy (NCT00183833), simultaneous PDGFR and VEGF inhibition to modify tumor angiogenesis (NCT00784446 and NCT01271166), and molecularly selected targeting of the mesenchymal CMS4 subtype (NCT02685046). Importantly, these studies have generally been early-phase, non-randomized, small or biomarker-driven investigations, and several were completed or terminated without establishing imatinib as an effective standard treatment for CRC. The clinical-trial experience therefore does not support the use of imatinib in unselected CRC patients. Instead, it supports a more selective therapeutic concept in which imatinib could potentially benefit molecularly defined tumors characterized by activation of susceptible pathways, particularly PDGFR/KIT signaling or mesenchymal/stromal programs, or could function as a rational component of combination therapies designed to overcome resistance to EGFR- or VEGF-directed treatment. The limited accrual and early termination of some studies also highlight an important translational challenge: despite substantial mechanistic evidence, identification of a sufficiently large and clinically well-defined CRC population dependent on an imatinib-sensitive pathway has remained difficult (Table 2).

## 6. Conclusions

Imatinib is a well-characterized, clinically established tyrosine kinase inhibitor with a pharmacological profile extending beyond ABL1/ABL2 to include KIT, PDGFR, DDR1 and, potentially, LCK. The evidence reviewed in this article indicates that these targets may contribute to distinct aspects of CRC biology, including tumor-cell proliferation and survival, migration and invasion, extracellular-matrix remodeling, angiogenesis, tumor–stroma interactions, treatment resistance and immune regulation. However, the biological contribution of individual targets is highly context-dependent, and the multi-target nature of imatinib makes it difficult to attribute its antitumor effects in CRC to a single molecular pathway.

Preclinical studies provide substantial support for the further investigation of imatinib in CRC. Antitumor effects have been reported in models characterized by KIT-dependent signaling, PDGFR-positive tumor stroma, and aberrant ABL1 or DDR1 activity. Importantly, several studies suggest that the potential value of imatinib may extend beyond direct cytotoxicity toward modulation of the tumor microenvironment, inhibition of invasion and metastasis, suppression of therapy-resistant or stem-like populations, and enhancement of the activity or delivery of other anticancer agents. The evidence concerning DDR1 and PDGFR is particularly relevant because these kinases may connect tumor-cell signaling with extracellular-matrix and stromal processes, providing a rationale for combination and microenvironment-directed therapeutic strategies rather than imatinib monotherapy.

Nevertheless, the available clinical evidence does not currently establish imatinib as an effective standard treatment for unselected CRC. Clinical studies have largely been small, early-phase, exploratory, or combination-based, and their findings are insufficient to determine whether imatinib provides a clinically meaningful benefit over contemporary standard therapies. The observed hematological and gastrointestinal toxicities, together with the complexity of combining imatinib with cytotoxic, antiangiogenic or molecularly targeted agents, further emphasize the need for careful dose selection and rational treatment design. Therefore, the translational potential of imatinib is unlikely to depend on nonspecific use in CRC and may instead lie in appropriately selected molecular and biological contexts.

Future research should consequently prioritize biomarker-guided patient selection, with particular attention to tumors characterized by KIT or PDGFR activation, stromal/mesenchymal features, and potentially increased DDR1 activity. CMS4 represents a biologically plausible, hypothesis-generating population for imatinib-based strategies, but it should not yet be considered a validated predictive biomarker of treatment response. Studies should also determine whether imatinib is most effective when administered in rational combinations with chemotherapy, antiangiogenic agents, immunotherapy, or other targeted inhibitors, and whether treatment sequencing influences therapeutic activity. The development of tumor-targeted formulations and delivery systems may provide an additional strategy to increase local drug exposure while limiting systemic toxicity and overcoming drug-efflux or other pharmacological barriers. Equally important will be the use of selective inhibitors, genetic approaches, and pharmacodynamic biomarkers to distinguish the contribution of individual imatinib targets from broader polypharmacological effects. Overall, imatinib should currently be regarded as a biologically plausible but clinically unvalidated therapeutic strategy in CRC, for which the next step is not empirical repurposing but mechanism-driven, biomarker-informed translational and clinical investigation.

## Figures and Tables

**Figure 1:**
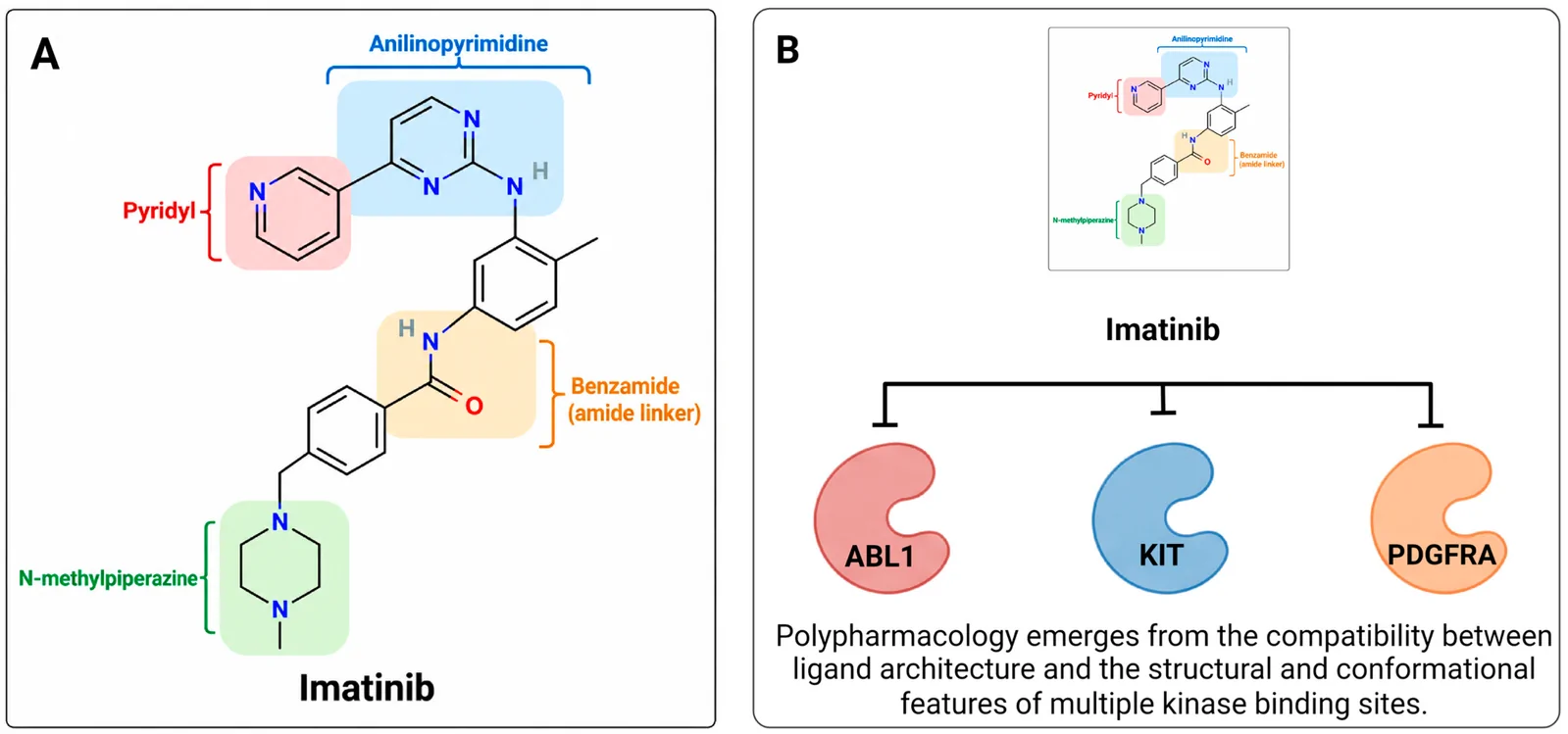
Pharmacophore features of imatinib and their relationship to type II kinase inhibition and polypharmacology. (**A**) Chemical structure of imatinib highlighting its key pharmacophore elements: the anilinopyrimidine moiety, pyridyl group, benzamide linker, and N-methylpiperazine substituent. (**B**) Schematic representation of imatinib’s polypharmacological activity against ABL1, KIT, and PDGFRA. Imatinib acts as a type II ATP-competitive kinase inhibitor by preferentially binding to the inactive DFG-out conformation of the kinase domain, extending from the ATP-binding site into an adjacent hydrophobic pocket. The interplay between its molecular architecture and the structural features of kinase-binding sites contributes to its multi-target activity. Created in BioRender. Kciuk, M. (2026) https://BioRender.com/br2k7ur (accessed on 27 October 2026).

**Figure 2:**
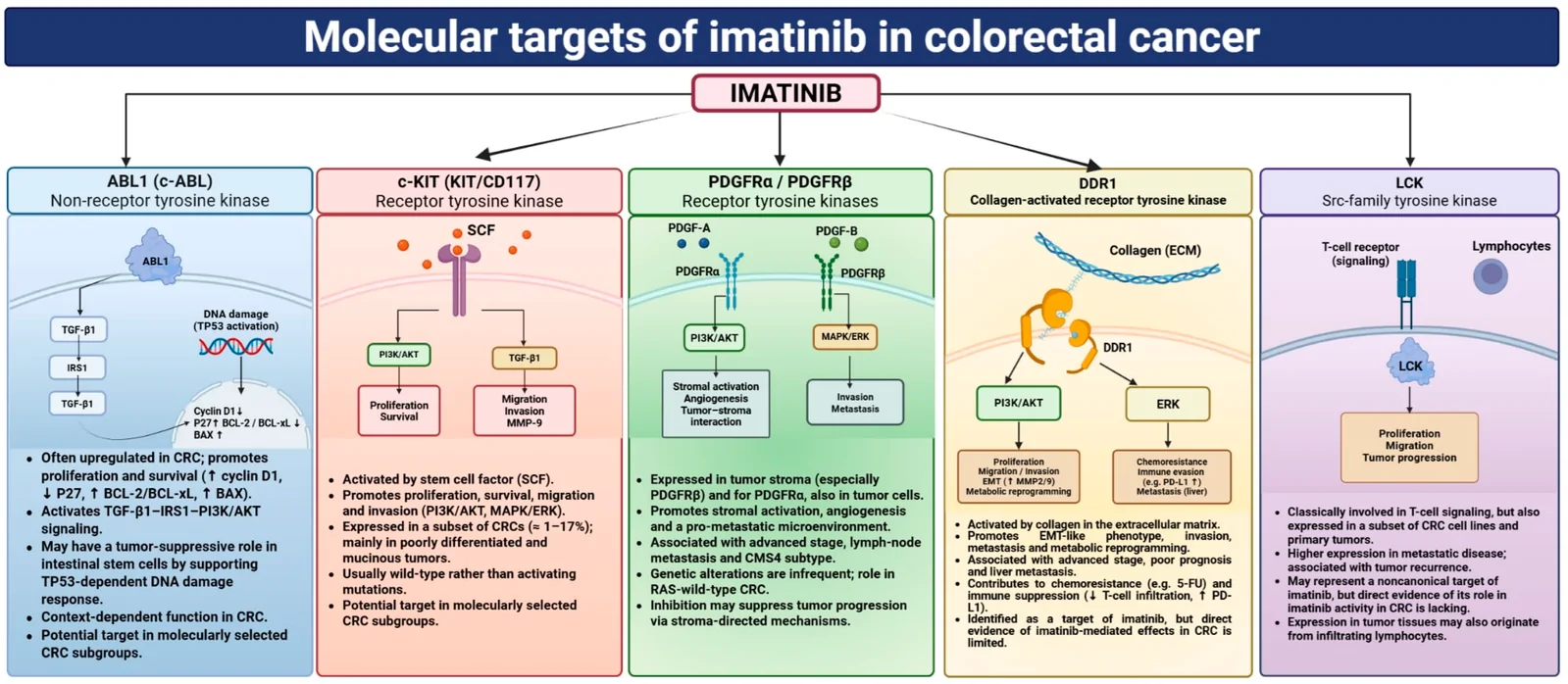
Molecular targets of imatinib in colorectal cancer (CRC). Schematic representation of the principal and potential molecular targets of imatinib relevant to CRC. Imatinib inhibits several tyrosine kinases that may contribute to tumor-cell signaling and tumor–microenvironment interactions. ABL1 (c-ABL), a non-receptor tyrosine kinase, is implicated in proliferation, survival, cytoskeletal dynamics, and cellular responses to DNA damage and stress, with reported interactions with TGF-β1–IRS1–PI3K/AKT signaling and regulation of cell-cycle and apoptosis-related proteins. c-KIT (KIT/CD117) is a receptor tyrosine kinase activated by SCF. Its downstream PI3K/AKT and MAPK/ERK pathways may promote CRC-cell proliferation, survival, migration, and invasion. PDGFR-α/PDGFR-β are receptor tyrosine kinases expressed particularly in tumor-associated stromal cells, including cancer-associated fibroblasts and pericytes, and their signaling contributes to stromal activation, angiogenesis, tumor–stroma interactions, invasion, and metastasis. DDR1, a collagen-activated receptor tyrosine kinase, links extracellular-matrix signaling to PI3K/AKT and ERK pathways and has been associated with proliferation, migration, invasion, EMT-like changes, chemoresistance, immune evasion, and liver metastasis. LCK, a SRC-family tyrosine kinase classically involved in T-cell receptor signaling, may also be expressed in subsets of CRC cells and tumor-associated lymphocytes. However, its contribution to the antitumor activity of imatinib in CRC remains insufficiently established. Overall, these targets illustrate the potentially pleiotropic effects of imatinib on tumor-cell proliferation and survival, invasion and metastasis, extracellular-matrix signaling, angiogenesis, chemoresistance, and the tumor microenvironment. The heterogeneous expression and context-dependent functions of these kinases should nevertheless be considered when interpreting the therapeutic effects of imatinib in CRC. For full names of proteins, see abbreviations section. Created in BioRender. Kciuk, M. (2026) https://BioRender.com/tkulrig (accessed on 27 October 2026).

**Figure 3:**
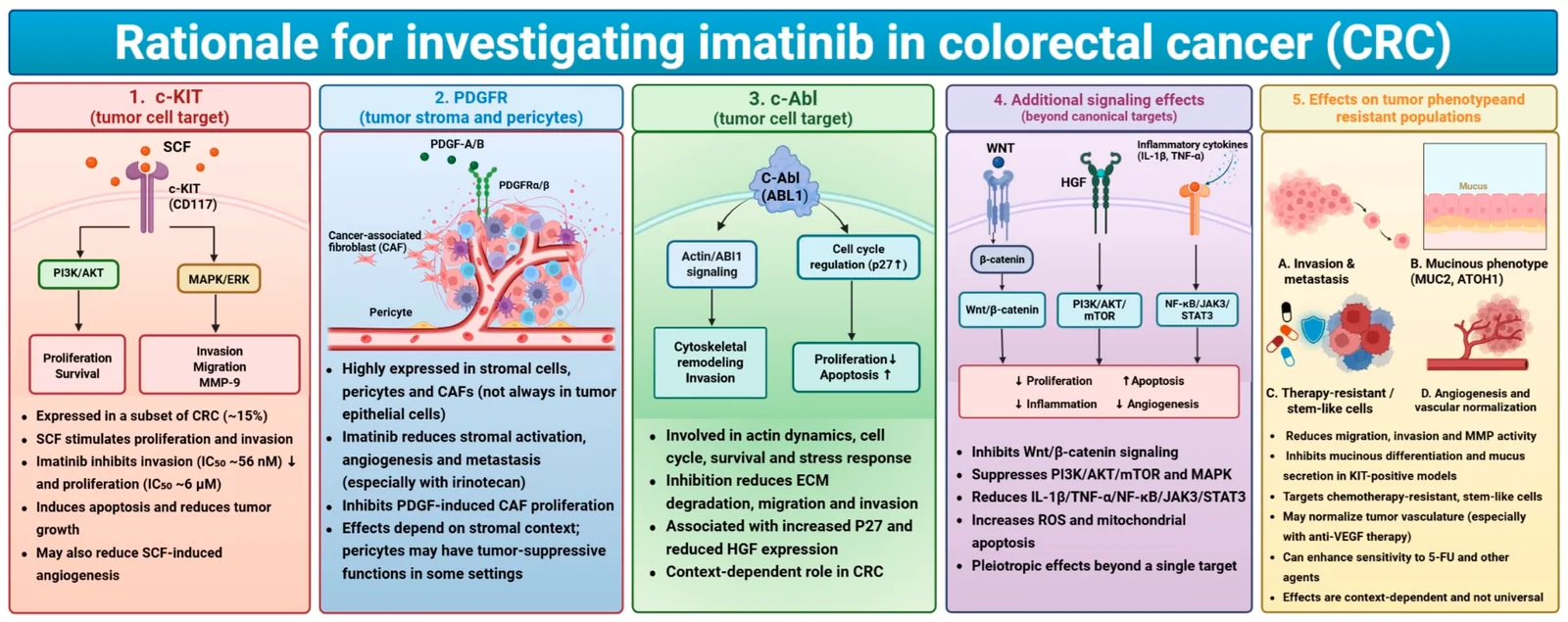
Rationale for investigating imatinib in CRC. Schematic overview of the proposed molecular and cellular mechanisms supporting the investigation of imatinib in CRC. (1) c-KIT (KIT/CD117) is expressed in a subset of CRC cells and can be activated by SCF, triggering PI3K/AKT and MAPK/ERK signaling associated with proliferation, survival, migration, and invasion. Imatinib-mediated inhibition of c-KIT may therefore reduce tumor-cell proliferation and invasion, promote apoptosis, and attenuate SCF-dependent angiogenic responses. (2) PDGFRα/β is predominantly expressed in tumor-associated stromal cells, including CAFs and pericytes, where PDGF signaling contributes to stromal activation, angiogenesis, tumor–stroma interactions, invasion, and metastasis. Imatinib may interfere with these stromal processes and thereby modify the tumor microenvironment. (3) ABL1 (c-Abl) contributes to actin dynamics, cytoskeletal remodeling, cell-cycle regulation, survival, and cellular responses to stress. Its inhibition by imatinib may increase P27 expression, reduce ECM degradation and migration, and promote apoptosis, although its role in CRC appears to be context-dependent. (4) Additional signaling effects beyond the canonical imatinib targets may involve modulation of Wnt/β-catenin, PI3K/AKT/mTOR, MAPK, NF-κB/JAK/STAT, and inflammatory signaling pathways, potentially resulting in reduced proliferation and inflammation, increased apoptosis, and impaired angiogenesis. (5) Effects on tumor phenotype and resistant populations include potential inhibition of invasion and MMP activity, attenuation of mucinous differentiation and mucus secretion in KIT-positive models, targeting of therapy-resistant or stem-like tumor-cell populations, and modulation of tumor vascularization. Collectively, these effects provide a mechanistic rationale for evaluating imatinib in combination with established anticancer therapies and in molecularly or biologically selected CRC subgroups. Importantly, the heterogeneous expression of these targets, the context-dependent functions of individual kinases, and the limited validation of non-canonical targets as predictive biomarkers indicate that the therapeutic relevance of imatinib in CRC requires appropriate molecular and biological stratification. For full names of proteins, see abbreviations section. Created in BioRender. Kciuk, M. (2026) https://BioRender.com/r0jstw9 (accessed on 27 October 2026).

**Figure 4:**
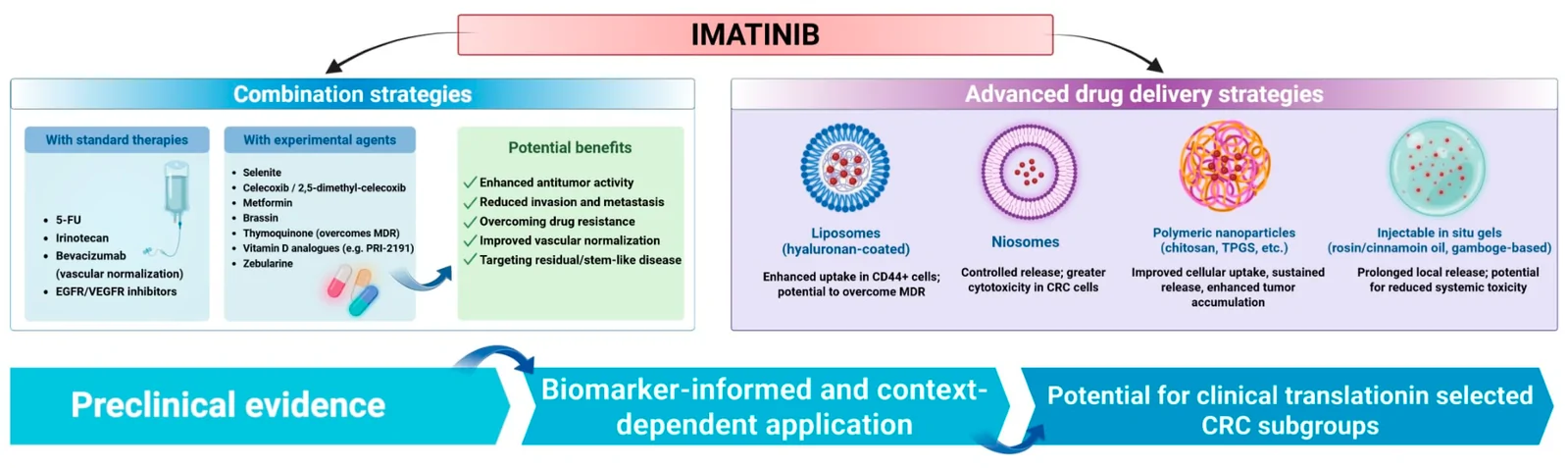
Translational strategies for imatinib in colorectal cancer. Created in BioRender. Kciuk, M. (2026) https://BioRender.com/dwfdubq (accessed on 27 October 2026).

**Table 1:** Preclinical evidence supporting the therapeutic potential of imatinib in colorectal cancer. The table summarizes experimental studies evaluating the antitumor activity of imatinib in colorectal cancer (CRC), including its effects on c-KIT, PDGFR and c-Abl signaling, tumor-cell proliferation, apoptosis, migration and invasion, tumor–stroma interactions, therapy-resistant and stem-like populations, and responses to combination treatment. Formulation-based strategies developed to improve imatinib delivery, cellular uptake, drug exposure and therapeutic efficacy are also included.

Target/Therapeutic Rationale	Principal Findings	Proposed Mechanism(s)	Major Implications/Limitations	Ref.
c-KIT/KIT–SCF signaling	c-KIT (~145 kDa) was detected in CRC cells. SCF stimulated invasion, whereas imatinib inhibited SCF-induced invasion with an IC_50_ of ~56 nM. Imatinib inhibited HT29 proliferation (IC_50_ ~6 μM), induced apoptosis and reduced tumor growth. SCF also exhibited proangiogenic activity.	Inhibition of SCF/c-KIT signaling; suppression of downstream PI3K/AKT and MAPK signaling	Provides early evidence for direct antitumor activity against KIT-positive CRC, but activity is unlikely to apply to unselected CRC.	PMID: 12208734
c-KIT/PI3K–AKT–MAPK	SCF increased proliferation and invasion. PI3K/AKT activation was strongly associated with both phenotypes, whereas p44/42 MAPK activation was particularly associated with invasion.	KIT → PI3K/AKT and MAPK/ERK signaling	Supports a mechanistic rationale for targeting KIT-dependent proliferative and invasive phenotypes.	PMID: 17410437
c-KIT-positive CRC subgroup	c-KIT overexpression occurred in ~15% of primary tumors and ~14% of lymph-node metastases. No hotspot KIT mutations were detected; many KIT-positive tumors carried *KRAS* and/or *BRAF* alterations. Imatinib inhibited proliferation and induced apoptosis irrespective of *KRAS*/*BRAF* status.	Pharmacological inhibition of KIT-dependent signaling despite absence of canonical activating KIT mutations	Suggests that KIT expression/function rather than KIT mutation may define a potentially sensitive subgroup.	PMID: 17487504
KIT/KITLG autocrine/paracrine signaling	KIT/KITLG expression was heterogeneous but present in a subset of tumors. KIT knockdown reduced proliferation and xenograft growth. Imatinib inhibited KIT-positive organoid and xenograft growth.	Autocrine/paracrine KIT–KITLG signaling promoting tumorigenicity	Stronger evidence for biomarker-guided KIT targeting; requires selection of KIT-positive tumors.	PMID: 26026391
PDGFR/tumor stroma	Imatinib + irinotecan markedly inhibited cecal and hepatic tumor growth, completely inhibited lymph-node metastasis, reduced PDGFRβ phosphorylation, stromal reaction, proliferation and pericyte coverage, and increased apoptosis. Effects were weaker/absent in subcutaneous tumors with limited stromal reaction.	Inhibition of stromal PDGFR signaling; disruption of tumor–stroma interactions	Supports a microenvironment-directed mechanism rather than exclusively direct tumor-cell cytotoxicity. Strongly dependent on tumor model and stromal context.	PMID: 17148668
PDGFR/cancer-associated fibroblasts (CAFs)	At 1 μM, imatinib abolished PDGF-induced CAF DNA synthesis. Higher concentrations reduced CAF viability but increased IL-6/IL-8 and modestly increased VEGF/bFGF release.	PDGFR inhibition in CAFs	Demonstrates potential stromal inhibition but also highlights paradoxical/pro-tumorigenic responses following strong stromal perturbation.	PMID: 17141949
PDGFR/vascular pericytes	PDGF-BB increased pericyte coverage but unexpectedly suppressed tumor growth. Imatinib-mediated PDGFR inhibition reduced pericyte content and increased tumor growth.	Loss of PDGFR-dependent vessel stabilization following pericyte inhibition	Important caution: PDGFR inhibition may be detrimental when PDGFR-positive stromal cells/pericytes exert tumor-suppressive or vessel-stabilizing functions.	PMID: 17641778
PDGFR/stromal fibroblast signaling	Imatinib inhibited CRC proliferation and antagonized fibroblast-mediated stimulation of HT29 proliferation. It also inhibited stromally induced MT1-MMP expression and pro-MMP2 activation.	PDGFR-dependent tumor–fibroblast communication and ECM remodeling	Provides a mechanistic explanation for anti-invasive effects of imatinib in stromally supported CRC.	PMID: 17721919
General antitumor/anti-invasive activity	Imatinib inhibited anchorage-independent colony formation, cell scattering and Matrigel invasion; increased apoptosis susceptibility; reduced BCL-2 and increased pro-apoptotic BCL-xS. It also sensitized cells to 5-FU.	Apoptotic signaling and suppression of invasive phenotype	Supports anti-invasive and chemosensitizing properties beyond simple growth inhibition.	PMID: 14726674
c-ABL/actin–ECM signaling	Inhibition of c-ABL-mediated ABI1 phosphorylation reduced ECM degradation, fibronectin attachment and invasion. ABI1 overexpression at invasive tumor edges was associated with tumor budding/infiltration.	c-ABL → ABI1 → actin/ECM remodeling	Provides a potential mechanistic link between imatinib-sensitive ABL signaling and CRC invasion.	PMID: 24913355
Cytoskeletal remodeling	Imatinib altered actin organization, caused actin aggregation and reduced polymerization, correlating with markedly reduced migration. At 10–50 μM, it induced phosphatidylserine externalization and caspase-3 activation.	Cytoskeletal disruption and apoptosis	Migration inhibition occurred alongside relatively high concentrations associated with apoptosis, complicating interpretation of migration-specific effects.	PMID: 21658383
Wnt/β-catenin signaling	STI571 inhibited constitutive and Wnt1-induced β-catenin activity and suppressed proliferation.	Cross-talk between tyrosine kinase signaling and Wnt/β-catenin	Suggests pathway-level effects that may extend beyond the canonical imatinib targets.	PMID: 12706873
HGF/cell-cycle regulation	Imatinib induced apoptosis, increased the G1 population and reduced S/G2-M populations; p27 increased and HGF expression decreased.	Suppression of HGF signaling and induction of cell-cycle inhibition	Supports growth arrest through mechanisms involving both survival signaling and cell-cycle control.	PMID: 27799652
Multi-pathway signaling/mitochondrial apoptosis	Imatinib interfered with PI3K/AKT/mTOR, WNT/β-catenin and MAPK pathways and reduced IL-1β/TNF-α/NF-κB/JAK3/STAT3 signaling. It increased Ca^2+^ and ROS, decreased mitochondrial membrane potential and promoted cytochrome-c release and caspase-3 activation.	Pleiotropic inhibition of oncogenic/inflammatory signaling and mitochondrial apoptotic signaling	Demonstrates that imatinib can produce broad cellular effects that cannot necessarily be attributed to one kinase.	PMID: 33022360
Therapy-resistant/CSC-like residual disease	Imatinib strongly suppressed clonogenicity of doxorubicin-refractory cells. It prevented tumor formation in 50% of mice receiving doxorubicin-pretreated cells and delayed tumor formation in remaining animals. No comparable prevention was observed against chemotherapy-naïve cells.	Targeting therapy-resistant/stem-like residual populations	Suggests that imatinib may have greater value as a sequential treatment against residual disease than as upfront cytoreductive monotherapy.	PMID: 26956644
5-FU-resistant/stemness phenotype	Imatinib reduced clonogenicity and downregulated stemness-associated genes. Combination with PRI-2191 produced greater suppression of stemness-related genes in HCT116/5-FU cells.	Suppression of cancer-cell plasticity and stemness	Supports investigation of imatinib in post-chemotherapy residual disease and recurrence prevention.	PMID: 30772447
Mucinous CRC/c-KIT	SCF/c-KIT signaling was strongly activated. Genetic or pharmacological KIT inhibition altered MUC2 and ATOH1 expression. c-KIT loss-of-function shifted tumors toward a non-mucinous phenotype. Imatinib suppressed ATOH1 after SCF/c-KIT stimulation.	KIT → AKT → inhibitory GSK3β phosphorylation → ATOH1 → MUC2	Suggests KIT involvement in mucinous differentiation and secretory biology, identifying a potential histopathological subgroup.	PMID: 29786668
c-KIT/PKCδ–MARCKS	SCF/c-KIT promoted mucus secretion. Imatinib reduced mucus secretion in mice, while manipulation of SCF/KIT altered MARCKS signaling.	KIT → PKCδ → MARCKS	Extends KIT relevance beyond proliferation to mucin production and tumor phenotype.	PMID: 30034943
Imatinib + 5-FU	Imatinib enhanced 5-FU-mediated growth inhibition, particularly in HT29 cells.	Complementary inhibition of survival/proliferation pathways	Provides a rationale for combination treatment, although optimal molecular selection remains undefined.	PMIDs: 14726674, 17721919
Imatinib + irinotecan	Combination strongly suppressed primary and metastatic tumor growth and completely prevented lymph-node metastasis in one model.	Direct tumor-cell effects plus stromal PDGFR inhibition	Supports combination with standard chemotherapy, particularly in tumors with substantial stromal involvement.	PMID: 17148668
EGFR/VEGFR + PDGFR blockade	AEE788 + imatinib + irinotecan produced the strongest inhibition of primary tumor growth and metastasis.	Simultaneous inhibition of tumor-cell EGFR/VEGFR and stromal PDGFR pathways	Supports simultaneous targeting of heterogeneous tumor and stromal signaling compartments.	PMID: 18324358
Imatinib + anti-VEGF therapy	Low-dose bevacizumab + imatinib achieved substantial tumor inhibition while promoting vascular maturation, reducing leakiness and hypoxia and increasing perfused/open vessels.	PDGFR inhibition-mediated vascular normalization combined with VEGF blockade	Provides a rationale for imatinib as a vascular-normalizing rather than solely cytotoxic agent.	PMID: 28141797
Imatinib + selenite	Synergistic cytotoxicity with increased caspase-3, reduced BCL-2, DNA fragmentation, S-phase arrest and inhibition of cytoprotective autophagy.	Enhanced apoptotic signaling and suppression of protective autophagy	Promising experimental combination; currently entirely preclinical.	PMID: 24930392
Imatinib + celecoxib	Combination produced greater loss of viability and increased caspase-3 activity with reduced COX-2 expression.	Concurrent modulation of tyrosine kinase and COX-2 signaling	Suggests pathway-combination strategy; requires validation in vivo.	PMID: 26950454
Imatinib + 2,5-dimethyl-celecoxib	Half-dose combination induced apoptosis comparable to full-dose imatinib and increased caspase-3 activity/expression.	Enhanced apoptotic signaling independent of classical COX-2 inhibition	Potential dose-sparing approach.	PMID: 28255316
Imatinib + metformin	Synergistic cytotoxicity, S/G2-M arrest, reduced cyclin B1 and pERK. Autophagy was induced and appeared predominantly cytoprotective; chloroquine further decreased viability.	ERK/cell-cycle suppression with cytoprotective autophagy	Suggests that simultaneous inhibition of protective autophagy may enhance imatinib activity.	PMID: 28785735
Imatinib + brassinin	Synergistic cytotoxicity and apoptosis, G0/G1 arrest and suppression of MMP-9 activity/expression.	Apoptotic and anti-invasive signaling	Supports a potential anti-metastatic combination strategy.	PMID: 30450754
Imatinib + thymoquinone	Thymoquinone markedly reduced imatinib IC_50_ from 105/72 μM to 7/5.5 μM with 10 μM thymoquinone and to 5.6/4.6 μM with 20 μM. Increased intracellular imatinib accumulation and synergistic interaction were observed.	Downregulation of *ABCB1*, *ABCG2* and hOCT1; increased intracellular drug exposure	Particularly relevant for overcoming MDR and improving intracellular imatinib availability.	PMID: 33783002
Imatinib + thymoquinone/autophagy–apoptosis	Combination induced apoptosis and autophagy, supported by apoptosis markers, AMPK/Par-4 signaling and colony-formation assays.	AMPK/Par-4-associated programmed cell death	Provides additional support for thymoquinone-mediated potentiation of imatinib.	PMID: 40143604
Imatinib + zebularine	Synergistic growth inhibition, increased oxidative stress, apoptosis/necrosis and S-phase arrest; reduced MMP-2/MMP-9, BCL-2 and EGFR.	Oxidative stress, apoptotic signaling and inhibition of invasion-associated pathways	Recent but entirely preclinical evidence requiring independent validation.	PMID: 42070100
TRAIL + imatinib—antagonistic interaction	Contrary to several positive combinations, imatinib reduced TRAIL-induced apoptosis.	Inhibition of c-ABL/p73/JNK/p38 stress-signaling cascade	Important negative finding demonstrating that imatinib is not universally synergistic and that drug sequencing/combination selection is critical.	PMID: 22462553
Hyaluronan-coated liposomal imatinib	Imatinib–deoxycholate conjugate-loaded liposomes were ~56.6 nm, with 99.1% entrapment; hyaluronan coating increased size to ~159 nm. Enhanced uptake occurred in CD44-positive cells and effects consistent with altered P-gp substrate trafficking were observed.	HA/CD44-mediated uptake and altered intracellular trafficking	Potential approach for MDR CRC; remains preclinical.	PMID: 25478964
Imatinib-loaded niosomes	Optimized niosomes were ~425 nm with 82.96% entrapment and controlled release. IC_50_ was 16.4-fold lower in HCT116 compared with free imatinib.	Improved drug delivery and controlled release	Strong HCT116-specific formulation evidence, but broader translational validation is needed.	PMID: 27544432
Chitosan-imatinib nanoparticles	Nanoparticles were ~208 nm, with 68.52% entrapment and 86.45% cumulative release. Enhanced colon-cell accumulation and greater controlled cytotoxicity than free imatinib were observed. Hemolysis was only 0.46%, with no evident histopathological damage.	Improved colon/tumor accumulation and controlled drug exposure	Promising localized/systemic delivery strategy; evidence remains preclinical.	PMID: 32070732
Chitosan–TPGS–imatinib nanoparticles	Enhanced cytotoxicity, apoptosis, uptake and migration inhibition relative to free imatinib; sustained release and preliminary pharmacokinetic/toxicity data were obtained.	Improved uptake, sustained release and mitochondrial/apoptotic effects	Potentially useful for overcoming poor cellular exposure; requires CRC-specific in vivo efficacy and safety validation.	PMID: 39567152

Abbreviations: 5-FU—5-fluorouracil; *ABCB1*—ATP-binding cassette subfamily B member 1; *ABCG2*—ATP-binding cassette subfamily G member 2; Abl/c-Abl—Abelson murine leukemia viral oncogene homolog 1; AKT—protein kinase B; AMPK—AMP-activated protein kinase; *BRAF*—B-Raf proto-oncogene; CAF—cancer-associated fibroblast; CRC—colorectal cancer; CSC—cancer stem cell; COX-2—cyclooxygenase-2; ECM—extracellular matrix; EGFR—epidermal growth factor receptor; ERK—extracellular signal-regulated kinase; GIST—gastrointestinal stromal tumor; GSK3β—glycogen synthase kinase 3 beta; HGF—hepatocyte growth factor; hOCT1—human organic cation transporter 1; IC_50_—half-maximal inhibitory concentration; IL—interleukin; KIT/c-KIT—KIT receptor tyrosine kinase (CD117); KITLG—KIT ligand; MAPK—mitogen-activated protein kinase; MARCKS–myristoylated alanine-rich protein kinase C substrate; MDR—multidrug resistance; MMP—matrix metalloproteinase; mTOR—mechanistic target of rapamycin; PDGFR—platelet-derived growth factor receptor; PI3K—phosphoinositide 3-kinase; PKCδ—protein kinase C delta; ROS—reactive oxygen species; SCF—stem cell factor; STAT3—signal transducer and activator of transcription 3; TGF-β—transforming growth factor beta; TNF-α—tumor necrosis factor alpha; VEGF—vascular endothelial growth factor; WNT—Wingless-related integration site.

**Table 2:** Clinical trials investigating imatinib mesylate-based therapeutic strategies in colorectal cancer. The table summarizes registered clinical studies evaluating imatinib, either as monotherapy or in combination with targeted agents and/or cytotoxic chemotherapy, including the study phase, ClinicalTrials.gov identifier, therapeutic agents, proposed mechanistic rationale, and recruitment status.

Official Study Title	NCT Number	Phase	Agents Used	Mechanistic Rationale	Study Status
Phase II Study Of Gleevec (Imatinib Mesylate Formerly Known As STI571 (NSC #716051) In Patients With Colorectal Cancer Stage IV	NCT00041340	Phase II	Imatinib mesylate (Gleevec)	Imatinib was investigated as a targeted therapy in metastatic CRC based on expression of imatinib-sensitive kinases, particularly c-KIT, ABL/ARG and PDGFR. The study also aimed to determine the biological effects of imatinib on the c-KIT/PDGFR system and downstream signaling, providing a biomarker-driven rationale for treatment.	Completed
Phase I/II Study of Gleevec^®^ Combined With Panitumumab (Vectibix^®^) in Patients Prescreened for C-kit/PDGFr Activated Pathways Using a Proteomic Based Assay	NCT00867334	Phase I/II	Imatinib + panitumumab; panitumumab monotherapy comparator	Designed to address resistance to EGFR blockade in metastatic CRC. Although *KRAS* wild-type status is required for panitumumab sensitivity, it is insufficient in many patients. Activation of alternative pathways, particularly PDGF/PDGFR and KIT, was proposed as a mechanism of resistance. Imatinib was therefore combined with panitumumab to inhibit potential bypass signaling. A proteomic assay was used to identify activated c-KIT/PDGFR pathways and phosphorylation of imatinib targets was assessed as a pharmacodynamic marker.	Completed
Phase I/II Study of Xeloda and Gleevec in Patients With Advanced Solid Tumors	NCT00183833	Phase I/II	Capecitabine (Xeloda) + imatinib (Gleevec)	Combined imatinib with capecitabine, a prodrug of 5-FU, to investigate whether inhibition of imatinib-sensitive tyrosine kinases could complement fluoropyrimidine-based chemotherapy. The principal objective was to establish the safe dose and dose-limiting toxicity of imatinib when administered with capecitabine in patients with advanced solid tumors, including CRC.	Completed
A Phase I/II Study of Capecitabine/Oxaliplatin (XELOX) in Combination With Bevacizumab and Imatinib as First-line Treatment of Patients With Stage IV Colorectal Cancer	NCT00784446	Phase I/II	Imatinib + capecitabine + oxaliplatin + bevacizumab	Based on simultaneous targeting of PDGFR- and VEGF-dependent angiogenic pathways. PDGFR signaling contributes to pericyte recruitment and vascular maturation; imatinib-mediated PDGFR inhibition was therefore hypothesized to disrupt tumor-vessel stabilization and increase sensitivity to VEGF blockade by bevacizumab. XELOX was incorporated as the cytotoxic backbone.	Completed
Imatinib treatment of poor prognosis mesenchymal-type primary colon cancer: a proof-of-concept study in the preoperative window period (ImPACCT)	NCT02685046	Phase II	Imatinib 400 mg/day	Specifically targeted CMS4 colon cancer, the mesenchymal/stromal CRC subtype associated with poor prognosis and increased recurrence risk. Preclinical data suggested that inhibition of PDGFR and KIT could suppress the aggressive mesenchymal phenotype. A 2-week preoperative treatment window enabled comparison of tumor tissue before and after imatinib to determine its molecular effects, including changes in the CMS4 phenotype.	Terminated—low accrual rate
Phase I, dose finding, multicentre study of Glivec^®^ in combination with mFOLFOX-Avastin^®^	NCT01271166	Phase I	Imatinib (Glivec) + mFOLFOX + bevacizumab (Avastin)	Investigated addition of imatinib to chemotherapy plus VEGF inhibition, principally exploiting its ability to inhibit PDGFR signaling. The rationale was that PDGFR inhibition could interfere with tumor stromal/vascular support and potentially complement bevacizumab-mediated VEGF blockade. The primary purpose was determination of the maximum tolerated dose (MTD) and dose-limiting toxicities.	Terminated

5-FU—5-fluorouracil; ABL—Abelson murine leukemia viral oncogene homolog 1/Abelson-related protein tyrosine kinases; CRC—colorectal cancer; CMS4—consensus molecular subtype 4; DLT—dose-limiting toxicity; EGFR—epidermal growth factor receptor; KIT (c-KIT)—KIT proto-oncogene receptor tyrosine kinase; mFOLFOX—modified folinic acid, fluorouracil, and oxaliplatin; MTD—maximum tolerated dose; NCT—National Clinical Trial identifier; PDGF—platelet-derived growth factor; PDGFR—platelet-derived growth factor receptor; RT-qPCR—reverse transcription quantitative polymerase chain reaction; VEGF—vascular endothelial growth factor; VEGFR—vascular endothelial growth factor receptor; XELOX—capecitabine plus oxaliplatin.

## Data Availability

No new data were created or analyzed in this study. Data sharing is not applicable to this article.

## Abbreviations

5-FU	5-fluorouracil
ABI1	Abelson interactor 1
ABL1/2	Abelson leukemia virus-associated protein ½
ABCB1	ATP-binding cassette subfamily B member 1
ABCG2	ATP-binding cassette subfamily G member 2
AKT	protein kinase B
AMPK	AMP-activated protein kinase
AOM	Azoxymethane
ATM	ataxia-telangiectasia mutated
ATP	adenosine triphosphate
BAX	BCL2-associated X protein
BCL2	B-cell lymphoma 2
BCR	breakpoint cluster region
BRAF	B-Raf proto-oncogene, serine/threonine kinase
bFGF	basic fibroblast growth factor
BCL-xL	B-cell lymphoma-extra large
BCL-xS	B-cell lymphoma-extra small
CAC	colitis-associated cancer
CAF	cancer-associated fibroblast
CD4/8/44/117	cluster of differentiation 4/8/44/117
CDKN1A (p21)	cyclin-dependent kinase inhibitor 1A (p21)
CHK1/2	checkpoint kinase ½
CMS4	consensus molecular subtype 4
COX-2	cyclooxygenase-2
CRC	colorectal cancer
CRISPR	clustered regularly interspaced short palindromic repeats
circRNA	circular RNA
CSC	cancer stem cell
CXCL10	C-X-C motif chemokine ligand 10
DDR1/2	discoidin domain receptor 1/2
DDX21	DEAD-box helicase 21
DNA-PKcs	DNA-dependent protein kinase catalytic subunit
DLT	dose-limiting toxicity
DSS	dextran sulfate sodium
ECM	extracellular matrix
EGFR	epidermal growth factor receptor
EMT	epithelial–mesenchymal transition
ERK	extracellular signal-regulated kinase
ETV4	ETS variant transcription factor 4
EZH2	enhancer of zeste homolog 2
FOLFOX	folinic acid, fluorouracil, and oxaliplatin
GIST	gastrointestinal stromal tumor
GNLY	granulysin
GSK-3	glycogen synthase kinase-3
GSK3β	glycogen synthase kinase 3 beta
H2AX	H2A.X histone family member
H3K27me3	trimethylated histone H3 lysine 27
HGF	hepatocyte growth factor
hOCT1	human organic cation transporter 1
IC50	half-maximal inhibitory concentration
ICAM1	intercellular adhesion molecule 1
IFP	interstitial fluid pressure
IL	interleukin
IRS1	insulin receptor substrate 1
JAG1	jagged canonical Notch ligand 1
JAK3	Janus kinase 3
JNK	c-Jun N-terminal kinase
KIT	KIT receptor tyrosine kinase
KITLG	KIT ligand
KRAS	KRAS proto-oncogene, GTPase
LAMP2	lysosomal-associated membrane protein 2
LCK	lymphocyte-specific protein tyrosine kinase
LGR5	leucine-rich repeat-containing G-protein-coupled receptor 5
MAPK	mitogen-activated protein kinase
MARCKS	myristoylated alanine-rich protein kinase C substrate
MCA	mucinous colorectal adenocarcinoma
MDR	multidrug resistance
miR	microRNA
mFOLFOX	modified folinic acid, fluorouracil, and oxaliplatin
MMP	matrix metalloproteinase
mRNA	messenger RNA
MSC	mesenchymal stem cell
MSS	microsatellite-stable
MT1	membrane-type 1
MTD	maximum tolerated dose
mTOR	mechanistic target of rapamycin
MUC2	mucin 2
NF-κB	nuclear factor kappa B
NSD2	nuclear receptor-binding SET domain protein 2
OS	overall survival
P73	tumor protein p73
P-gp	P-glycoprotein
PAR-4	prostate apoptosis response protein 4
PD-1	programmed cell death protein 1
PD-L1	programmed death-ligand 1
PDGF	platelet-derived growth factor
PDGFR	platelet-derived growth factor receptor
PDGFRα	platelet-derived growth factor receptor alpha
PDGFRβ	platelet-derived growth factor receptor beta
PET/CT	positron emission tomography/computed tomography
PFS	progression-free survival
PI3K	phosphoinositide 3-kinase
PKCδ	protein kinase C delta
PTF	water-perfusible tissue fraction
CDKN1B (p27)	cyclin-dependent kinase inhibitor 1B (p27)
RAS	rat sarcoma
ROS	reactive oxygen species
RSK	ribosomal S6 kinase
RT-qPCR	reverse transcription quantitative polymerase chain reaction
RTK	receptor tyrosine kinase
SCF	stem cell factor
SLC6A6	solute carrier family 6 member 6
siRNA	small interfering RNA
SRC	sarcoma proto-oncogene
STAT3	signal transducer and activator of transcription 3
TCGA	The Cancer Genome Atlas
TGF-β	transforming growth factor beta
TNF-α	tumor necrosis factor alpha
TNM	tumor–node–metastasis
TPGS	D-α-tocopheryl polyethylene glycol 1000 succinate
TRAIL	TNF-related apoptosis-inducing ligand
TSC	tuberous sclerosis complex
UICC	Union for International Cancer Control
VCAM1	vascular cell adhesion molecule 1
VEGF	vascular endothelial growth factor
VEGFR	vascular endothelial growth factor receptor
WNK1	WNK lysine-deficient protein kinase 1
WNT	Wingless-related integration site
XELOX	capecitabine plus oxaliplatin
